# S_N_2 versus S_N_2′ Competition

**DOI:** 10.1021/acs.joc.2c00527

**Published:** 2022-06-24

**Authors:** Thomas Hansen, Pascal Vermeeren, Lea de Jong, F. Matthias Bickelhaupt, Trevor A. Hamlin

**Affiliations:** † Department of Theoretical Chemistry, Amsterdam Institute of Molecular and Life Sciences (AIMMS), Amsterdam Center for Multiscale Modeling (ACMM), Vrije Universiteit Amsterdam, De Boelelaan 1083, 1081 HV Amsterdam, The Netherlands; ‡ Leiden Institute of Chemistry, Leiden University, Einsteinweg 55, 2333 CC Leiden, The Netherlands; § Departament de Química Inorgànica i Orgànica & IQTCUB, Universitat de Barcelona, 08028 Barcelona, Spain; ∥ Institute for Molecules and Materials (IMM), Radboud University, Heyendaalseweg 135, 6525 AJ Nijmegen, The Netherlands

## Abstract

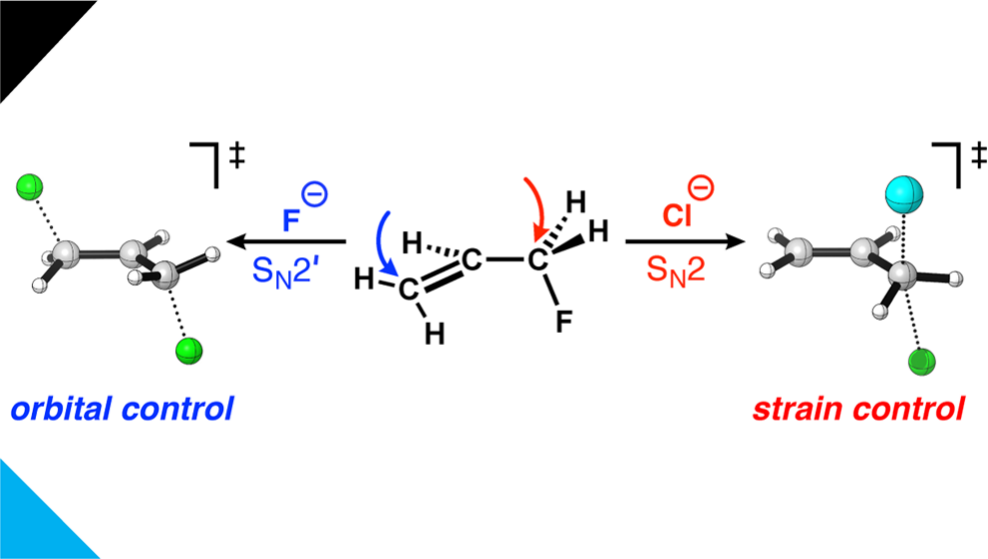

We have quantum chemically explored the competition between the S_N_2 and S_N_2′ pathways for X^–^ + H_2_C=CHCH_2_Y (X, Y = F, Cl, Br, I) using a combined relativistic density functional theory and coupled-cluster theory approach. Bimolecular nucleophilic substitution reactions at allylic systems, i.e., C^γ^=C^β^–C^α^–Y, bearing a leaving-group at the α-position, proceed either via a direct attack at the α-carbon (S_N_2) or via an attack at the γ-carbon, involving a concerted allylic rearrangement (S_N_2′), in both cases leading to the expulsion of the leaving-group. Herein, we provide a physically sound model to rationalize under which circumstances a nucleophile will follow either the aliphatic S_N_2 or allylic S_N_2′ pathway. Our activation strain analyses expose the underlying physical factors that steer the S_N_2/S_N_2′ competition and, again, demonstrate that the concepts of a reaction’s “characteristic distortivity” and “transition state acidity” provide explanations and design tools for understanding and predicting reactivity trends in organic synthesis.

## Introduction

**Scheme 1:**
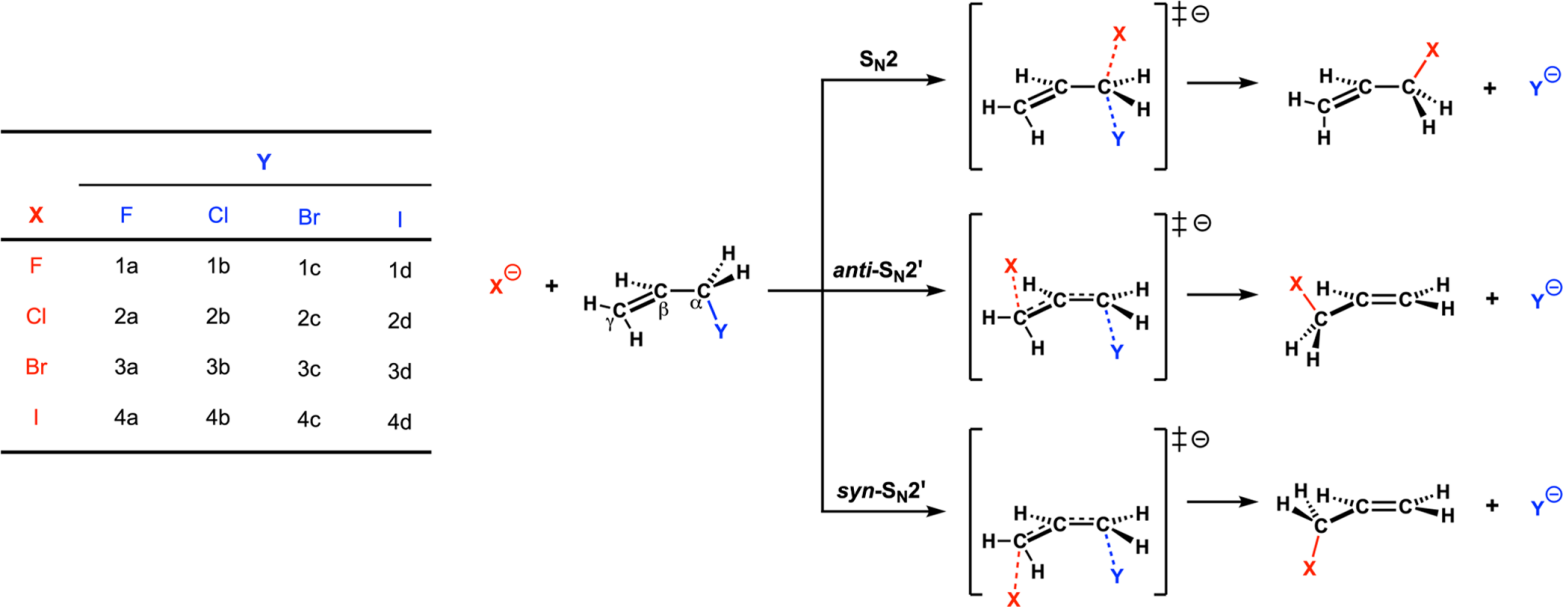
S_N_2 and S_N_2′ Pathways for Reactants X^–^ + H_2_C=CHCH_2_Y (X, Y = F, Cl, Br, I)

Bimolecular nucleophilic substitution reactions at allylic systems bearing a leaving-group at the α-position give rise to a competition between the aliphatic S_N_2 and allylic S_N_2′ reaction channels (Scheme 1). For this class of substrates, the nucleophile, i.e., Lewis base, can directly attack either the α-carbon (S_N_2) or the γ-carbon involving a concerted allylic rearrangement (S_N_2′, also known as S_N_2 prime).^1^ This intrinsic competition can result in unwanted side products and hampers the applicability of these transformations in synthetic chemistry. Nevertheless, in the last years, the S_N_2′ reaction has advanced into an important member of the chemical toolbox of synthetic chemists. Especially, the copper-catalyzed S_N_2′ reaction has become a key synthetic methodology to forge new C–C bonds with good regio- and stereoselectivity.^2,3^

Both experimental^4^ and theoretical studies^5^ have provided insights into the S_N_2/S_N_2′ competition. In general, the aliphatic S_N_2 pathway is favored; however, the allylic S_N_2′ can become dominant when an attack on the α-position is sterically retarded. Following this, the use of model substrates such as H_2_C=CHCR_2_Y (e.g., R = alkyl group) presents an almost exclusive preference for the S_N_2′ reaction.^6^ Similarly, allylic rearrangements of propargylic systems (C^γ^≡C^β^–C^α^–Y) conveniently furnish allenes.^7^ Despite these advances, the physical phenomena governing the preference of the S_N_2/S_N_2′ reaction channels are currently lacking in the literature. This ultimately thwarts the judicious tuning of the reactivity toward the desired pathway.

To this end, we have analyzed the reaction profiles of the S_N_2, *anti*-S_N_2′, and *syn*-S_N_2′ reaction pathways of X^–^ + H_2_C=CHCH_2_Y with X, Y = F, Cl, Br, I, using relativistic density functional theory (DFT). We have also computed DLPNO-CCSD(T) reference data that confirm the reliability of our DFT approach. The archetypal substrate H_2_C=CHCH_2_Y is an ideal model system to probe the competition between S_N_2/S_N_2′ and derive the intrinsic underlying physical factors and properties of the system that steer this competition. This study equips us with a systematic overview of reactivity trends over a wide range of reactivities and pathways, which can be extended to any substrate where the allylic system and the leaving-group are electronically coupled. The activation strain model (ASM)^8^ of reactivity in conjunction with Kohn–Sham molecular orbital (KS–MO) theory^9,10^ were employed to pinpoint the physical phenomena that control the competition between the aliphatic S_N_2 and allylic S_N_2′ pathway of the aforementioned reaction. In line with our previous work on the S_N_2/E2 reaction,^11^ the S_N_2/S_N_2′ competition could be traced back to (i) the “characteristic distortivity” of the substrate, which is directly connected with the “transition state acidity” for each specific reaction pathway; (ii) the strength of the nucleophile, i.e., Lewis base, which enters into an acid–base-like interaction with the substrate; and (iii) the leaving-group capacity.

## Results and Discussion

### General Trends in Reactivity

**Figure 1:**
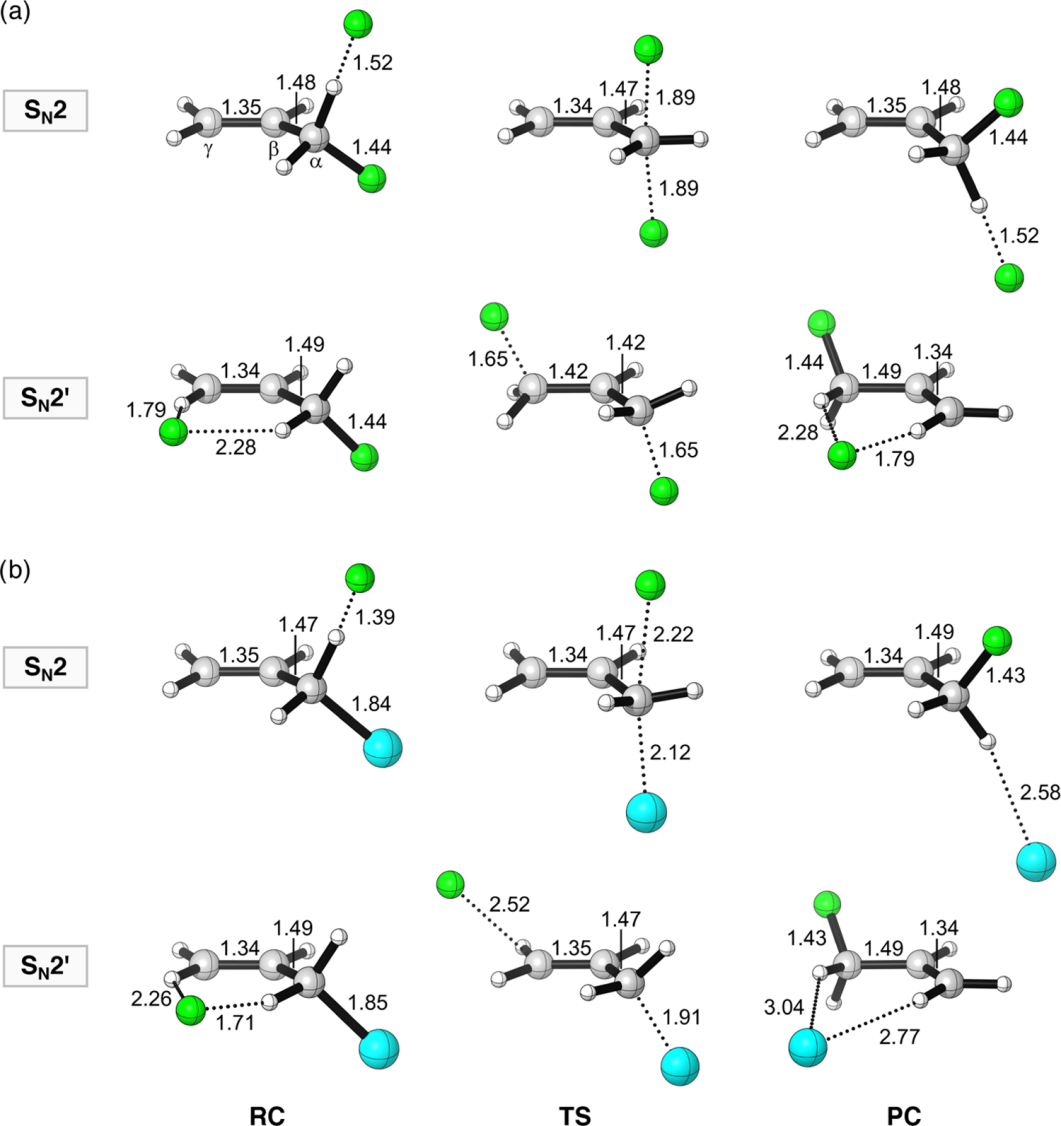
Structures (in Å) of stationary points along the aliphatic S_N_2 and allylic *anti*-S_N_2′ pathways of (a) F^–^ + H_2_C=CHCH_2_F and (b) F^–^ + H_2_C=CHCH_2_Cl, computed at ZORA-OLYP/QZ4P. Atom colors: carbon (gray), hydrogen (white), fluorine (green), and chlorine (cyan).

The computed reaction profiles of all the S_N_2, *anti*-S_N_2, and *syn*-S_N_2′ reactions of X^–^ + H_2_C=CHCH_2_Y with X, Y = F, Cl, Br, and I at ZORA-M06-2X/QZ4P//ZORA-OLYP/QZ4P and (TightPNO)DLPNO-CCSD(T)/CBS(3,4/def2)//ZORA-OLYP/QZ4P are collected in Table 1. In all cases, the reaction proceeds via a double-well potential energy surface (PES), going from the reactant complex (RC) through a transition state (TS) towards the product complex (PC), which may ultimately dissociate into the separated products (P). Structural data of the stationary points for two representative reactions are shown in Figure 1. Note that the overall activation energy Δ*E*^‡^, that is, the energy difference between the TS and the infinitely separated reactants (X^–^ and H_2_C=CHCH_2_Y), can be negative if a substantially stabilized reactant complex is formed. For a more detailed discussion on the various types of reaction potential energy surfaces, see, for example, ref (12). Importantly, the computed trends in reactivity at ZORA-M06-2X/QZ4P//ZORA-OLYP/QZ4P agree well with those calculated at the more accurate (TightPNO)DLPNO-CCSD(T)/CBS(3,4/def2)//ZORA-OLYP/QZ4P level (see Table 1).

**Table 1:** Energies Relative to the Reactants for All Stationary Points of the S_N_2, *Anti*-S_N_2′, and *Syn*-S_N_2′ Pathways Following X^–^ + H_2_C=CHCH_2_Y → H_2_C=CHCH_2_X + Y^–^ in kcal mol^–1 ^a

Y									
X^–^	**species**	**F (a)**		**Cl (b)**		**Br (c)**		**I (d)**	
F^–^ (1)	RC-S_N_2	–17.0	(−16.5)	–21.3	(−20.5)	–22.4	(−21.6)	–24.0	(−22.8)
	RC-S_N_2′	–21.2	(−20.9)	–23.7	(−23.6)	–24.5	(−24.2)	–25.1	(−24.8)
	TS-S_N_2	0.7	(−1.2)	–14.0	(−13.8)	–18.2	(−17.0)	–18.8	(−17.7)
	TS-*anti*-S_N_2′	–6.8	(−4.1)	–13.0	(−11.8)	–13.4	(−11.5)	–13.5	(−11.7)
	TS-*syn*-S_N_2′	–3.9	(−3.8)	–10.2	(−8.8)	–10.3	(−7.9)	–10.3	(−7.6)
	PC-S_N_2	–17.0	(−16.5)	–45.9	(−44.8)	–53.6	(−52.3)	–60.2	(−55.1)
	PC-S_N_2′	–21.2	(−20.9)	–48.5	(−47.2)	–55.8	(−54.2)	–61.9	(−56.7)
	P	0.0	(0.0)	–38.1	(−36.4)	–46.9	(−42.2)	–54.9	(−49.3)
Cl^–^ (2)	RC-S_N_2	–7.8	(−8.4)	–9.8	(−10.5)	–10.4	(−11.4)	–11.0	(−11.8)
	RC-S_N_2′	–10.4	(−10.8)	–12.0	(−12.4)	–12.3	(−12.8)	–12.6	(−13.2)
	TS-S_N_2	24.1	(22.6)	4.5	(4.9)	–0.8	(1.5)	–4.3	(−1.7)
	TS-*anti*-S_N_2′	25.1	(24.6)	7.9	(10.6)	2.4	(7.2)	–1.3	(2.8)
	TS-*syn*-S_N_2′	27.9	(27.6)	10.6	(13.8)	5.4	(10.1)	2.1	(6.0)
	PC-S_N_2	16.8	(16.0)	–9.8	(−10.5)	–17.3	(−15.3)	–23.6	(−20.2)
	PC-S_N_2′	14.4	(12.9)	–12.0	(−12.4)	–19.1	(−16.9)	–25.0	(−21.4)
	P	38.1	(36.4)	0.0	(0.0)	–8.8	(−5.7)	–16.8	(−12.9)
Br^–^ (3)	RC-S_N_2	–6.7	(−7.3)	–8.5	(−9.5)	–10.3	(−11.1)	–10.5	(−11.7)
	RC-S_N_2′	–8.9	(−9.0)	–10.3	(−11.2)	–10.4	(−11.5)	–10.8	(−11.8)
	TS-S_N_2	28.7	(25.2)	8.0	(7.2)	2.0	(3.8)	–1.7	(0.6)
	TS-*anti*-S_N_2′	33.5	(30.7)	11.1	(12.9)	5.1	(9.2)	1.2	(5.9)
	TS-*syn*-S_N_2′	36.6	(34.2)	14.2	(15.9)	8.2	(12.4)	4.5	(8.6)
	PC-S_N_2	24.5	(20.6)	–1.5	(−5.4)	–10.4	(−11.4)	–15.1	(−14.9)
	PC-S_N_2′	22.4	(18.0)	–3.6	(−7.0)	–10.4	(−11.5)	–16.4	(−15.9)
	P	46.9	(42.2)	8.8	(5.7)	0.0	(0.0)	–8.0	(−7.1)
I^–^ (4)	RC-S_N_2	–5.3	(−5.8)	–6.7	(−7.3)	–7.1	(−7.7)	–7.4	(−8.2)
	RC-S_N_2′	–7.0	(−7.4)	–8.2	(−8.5)	–8.3	(−8.8)	–8.5	(−8.9)
	TS-S_N_2	36.2	(31.6)	12.5	(11.1)	6.4	(7.8)	2.2	(4.9)
	TS-*anti*-S_N_2′	41.4	(37.6)	15.6	(15.7)	9.3	(13.0)	5.0	(9.3)
	TS-*syn*-S_N_2′	44.7	(41.7)	18.9	(18.9)	12.5	(15.7)	7.3	(11.0)
	PC-S_N_2	30.9	(26.5)	5.9	(1.1)	–1.4	(−3.5)	–7.4	(−8.2)
	PC-S_N_2′	29.9	(24.5)	4.2	(−0.4)	–2.8	(−4.7)	–8.5	(−8.9)
	P	54.9	(49.3)	16.8	(12.9)	8.0	(7.1)	0.0	(0.0)

a Computed at ZORA-M06-2X/QZ4P//ZORA-OLYP/QZ4P and (TightPNO)DLPNO-CCSD(T)/CBS(3,4/def2)//ZORA-OLYP/QZ4P in parentheses (see Scheme 1 for designation of species).

Several interesting reactivity trends can be derived from the reaction profiles (Table 1). In the first place, in line with experimental findings,^4^ for most of the studied systems, except for X, Y = F, there is a preference for the aliphatic S_N_2 pathway (see also Figure 6a).^4^ Second, of the two possible allylic S_N_2′ pathways, that is *anti*-S_N_2′ and *syn*-S_N_2′, the *anti*-S_N_2′ reaction pathway consistently goes with a lower activation energy than the *syn*-S_N_2′ pathway (ΔΔ*E*^‡^ = −2.3 to −3.4 kcal mol^–1^ for *anti*-S_N_2′ relative to *syn*-S_N_2′ using DFT) regardless of the nucleophile and leaving-group combination. As such, we will limit further discussion solely to the comparison between the *anti*-S_N_2′ and S_N_2 reaction pathways. Third, the activation energy of all nucleophilic substitution reactions (S_N_2, *anti*-S_N_2′, and *syn*-S_N_2′) increases when a weaker, i.e., less basic, anionic nucleophile X^–^ is used going from F^–^ to Cl^–^ to Br^–^ to I^–^. For example, the activation energy for the aliphatic S_N_2 pathway increases going from Δ*E*^‡^ = +0.7 to +24.1 to +28.7 to +36.2 kcal mol^–1^ upon going from X^–^ = F^–^ to Cl^–^ to Br^–^ to I^–^, when Y = F is the leaving-group. This is a manifestation of the reduced intrinsic nucleophilicity along this series.^11^ Interestingly, our computations reveal that along the same series, the *anti*-S_N_2′ activation energy rises to a larger extent than the S_N_2 activation energy, inducing a switch in the preferred reaction pathway from *anti*-S_N_2′ when the nucleophile is F^–^ to S_N_2 for nucleophiles Cl^–^, Br^–^, and I^–^ (ΔΔ*E*^‡^ = +7.6, −1.1, −4.8, −5.3 kcal mol^–1^ for S_N_2 relative to *anti*-S_N_2′). Thus, in this series, the *anti*-S_N_2′ dominates for the more basic halide X^–^ = F^–^, with an activation energy that is 7.6 kcal mol^–1^ lower than that of the S_N_2 pathway, whereas the S_N_2 pathway prevails for the heavier, less basic halides, X^–^ = Cl^–^, Br^–^, and I^–^. For the latter three nucleophiles, regardless of the leaving-group Y, the S_N_2 pathway is always preferred, which is a direct result of their lower basicity (*vide infra*).

On the other hand, for any nucleophile X^–^, varying the leaving-group along Y = F, Cl, Br, and I significantly lowers all nucleophilic substitution activation energies. For example, the activation energy for the S_N_2 pathway decreases from Δ*E*^‡^ = +0.7 to −14.0 to −18.2 to −18.8 kcal mol^–1^ along Y = F, Cl, Br, and I, respectively, using nucleophile X = F^–^. Importantly, along the same series, the most favorable reaction pathway switches from *anti*-S_N_2′ for Y = F to S_N_2 for Y = Cl, Br, and I, because the activation energy of the latter reaction decreases more rapidly than that of the former reaction (e.g., ΔΔ*E*^‡^ = +7.6, −1.1, −4.8, −5.3 kcal mol^–1^ for S_N_2 relative to *anti*-S_N_2′; see Table 1). In the next section, the origin of these reactivity trends is further analyzed and rationalized on the basis of the activation strain model (ASM)^8^ of reactivity in combination with quantitative Kohn–Sham molecular orbital (KS–MO)^9,10^ theory.

### Activation Strain Analyses

**Figure 2:**
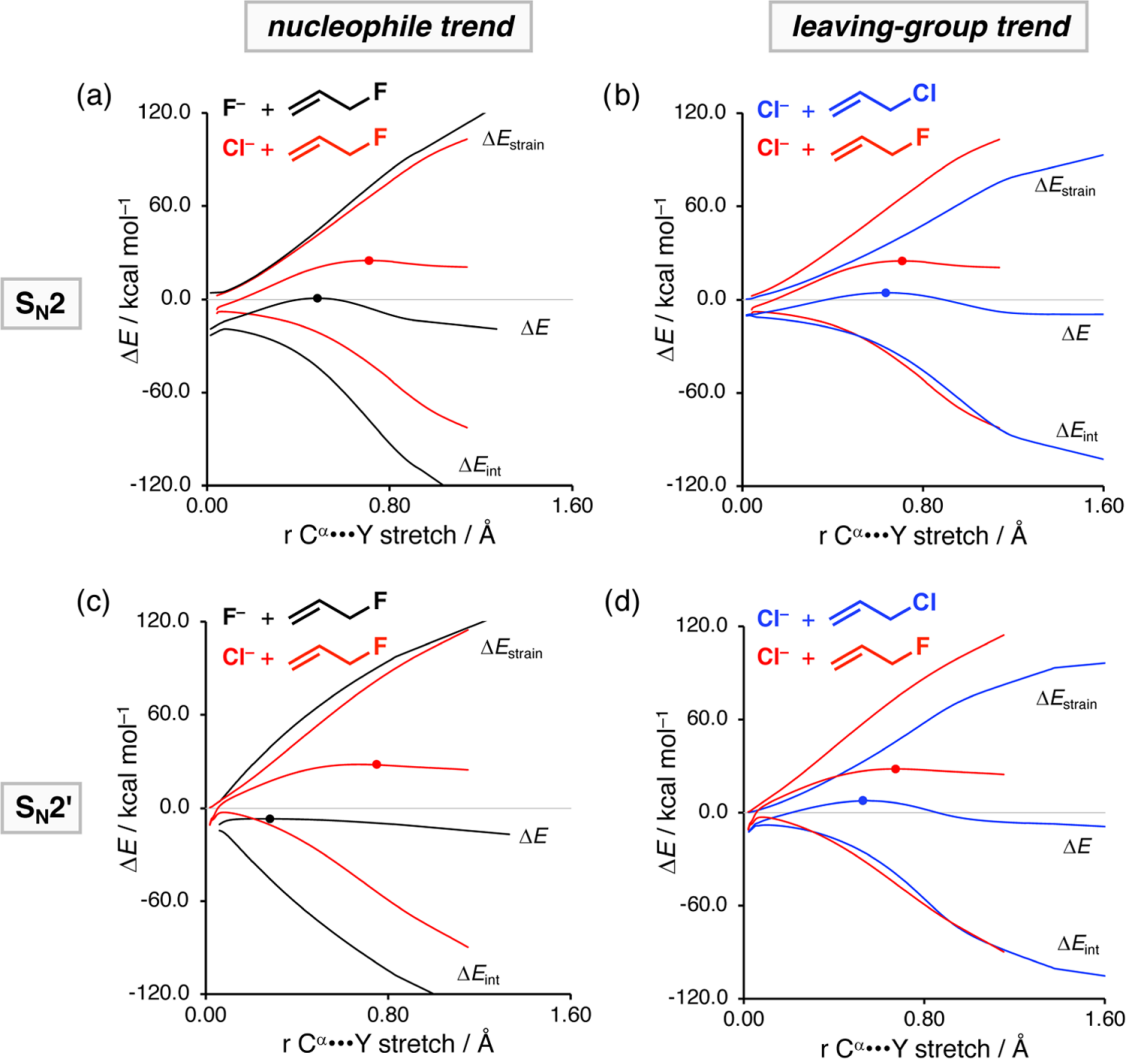
Activation strain analysis of the S_N_2 and *anti*-S_N_2′ reactions of X^–^ and H_2_C=CHCH_2_Y with X, Y = F, Cl, along the IRC projected on the C^α^···Cl bond stretch. The (a, c) left column shows the influence of the nucleophile on the PES, whereas the (b, d) right column shows the impact of the leaving-group variation. Transition states are indicated with dots. Computed at ZORA-M06-2X/QZ4P//ZORA-OLYP/QZ4P.

**Figure 3:**
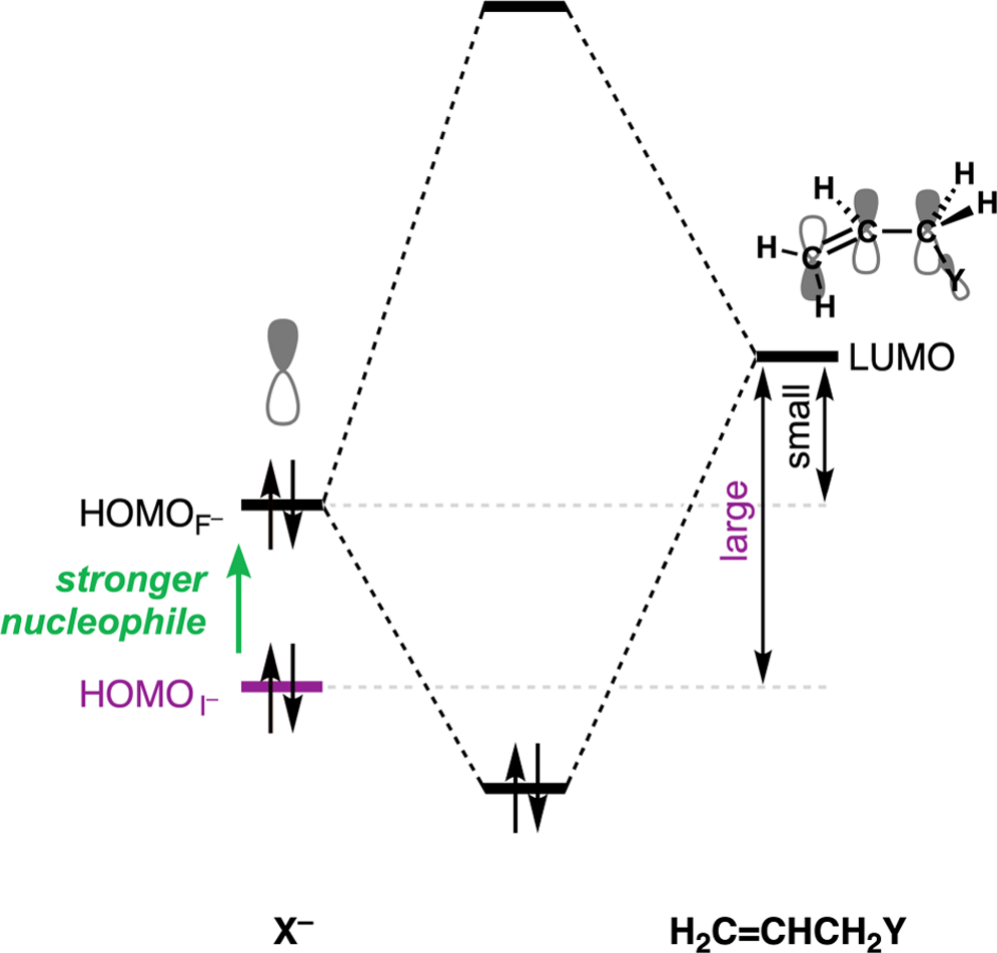
Schematic orbital interaction diagram between the filled n*p* HOMO of X^–^ (X = I^–^ to F^–^) and the LUMO of H_2_C=CHCH_2_Y.

**Figure 4:**
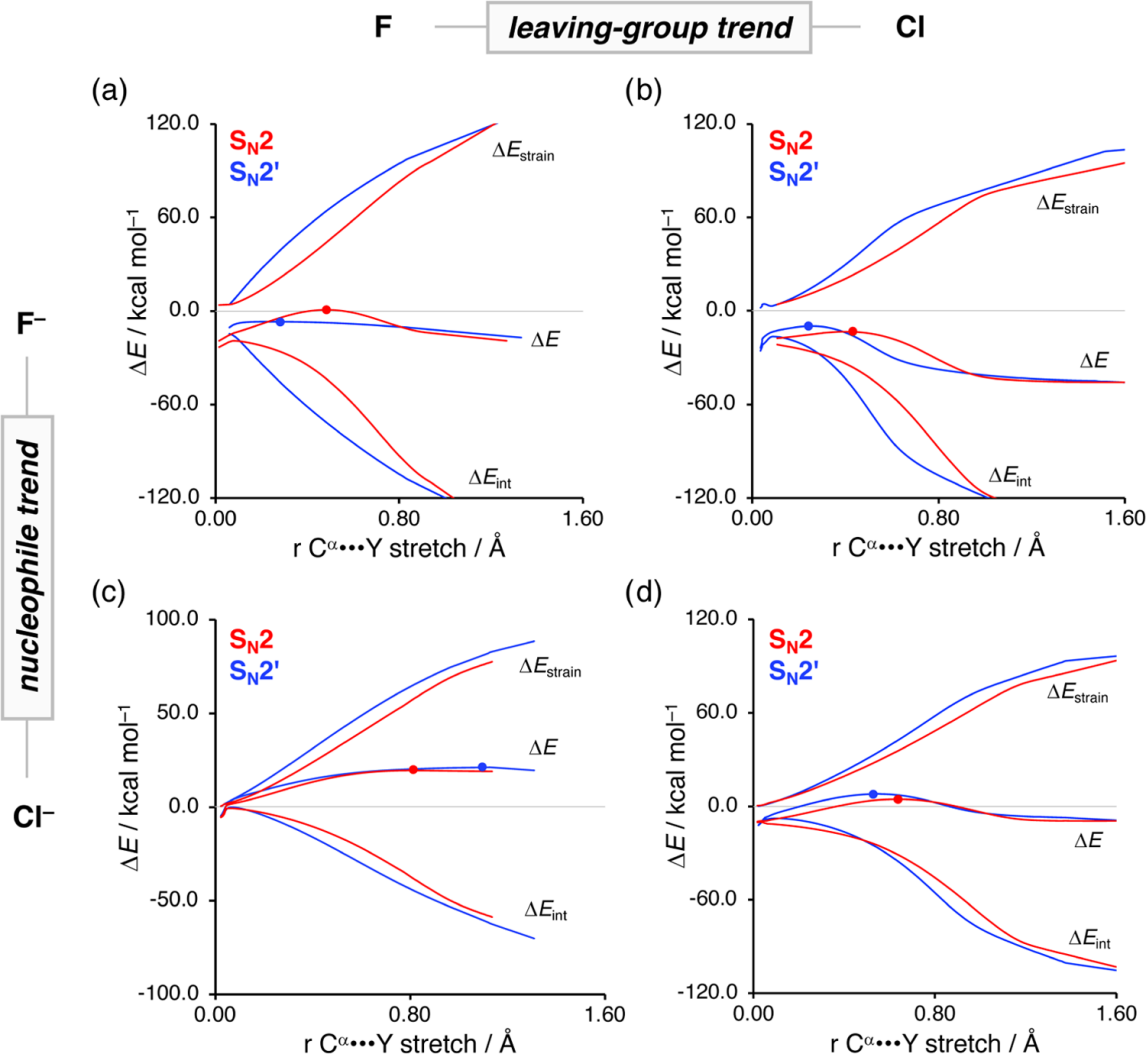
Activation strain analysis of the competition between S_N_2 (red) and *anti*-S_N_2′ (blue) reactions of anionic X^–^ and H_2_C=CHCH_2_Y with X, Y = F, Cl, along the IRC projected on the C^α^···Cl bond stretch. (a–c, and b–d) Trends in the vertical direction show the impact of the different nucleophiles on the competition, whereas (a, b, and c, d) trends in the horizontal direction show the influence of leaving-group variation. Transition states are indicated with dots. Computed at ZORA-M06-2X/QZ4P//ZORA-OLYP/QZ4P.

**Figure 5:**
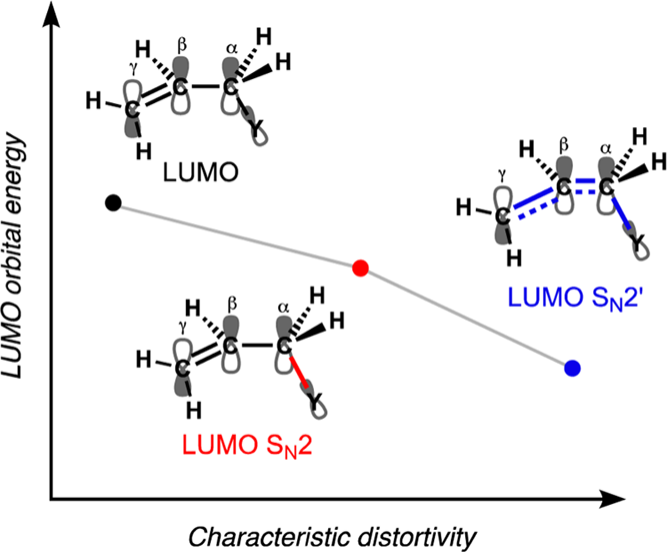
Schematic representation of the relation between the LUMO energy of the substrate and the characteristic distortivity. See Figure S2 for quantitative data on the relationship between characteristic distortivity and electronic structure.

The results emerging from utilizing the activation strain model (ASM) of reactivity for the representative S_N_2 and *anti*-S_N_2′ reactions of X^–^ and H_2_C=CHCH_2_Y (X, Y = F, Cl) are summarized in Figures 2–5 (see the Supporting Information Figure S1 for all activation strain diagrams). The ASM is a fragment-based approach in which the potential energy surface (PES) can be described with respect to, and understood in terms of, the characteristics of the reactants, i.e., the nucleophile and substrate. This analysis method decomposes the total electronic energy (Δ*E*) into two distinct terms, that is, the strain energy (Δ*E*_strain_) and the interaction energy (Δ*E*_int_). The strain energy is the penalty that needs to be paid to deform the individual reactants to react and the interaction energy accounts for all mutual interactions between the deformed reactants along the entire reaction coordinate, in this case, defined as the IRC projection onto the C^α^···Y stretch.^11,13^ This is a critical reaction coordinate that is intimately connected to the progress of nucleophilic substitution reactions on going from the reactant complex to the transition state to the product complex.

In Figure 2, we show how changing the anionic nucleophile X^–^, panels (a) and (c), and the leaving-group Y, panels (b) and (d), affect the reactivity of both the aliphatic S_N_2 and allylic S_N_2′ pathways. Changing the nucleophile from F^–^ to Cl^–^ leads, in line with its decreased basicity, to a significant loss of stabilizing interaction energy between the nucleophile and substrate, and hence results in an increase of the activation energy (Figure 2a,c). The strain energy, in contrast, is hardly affected by changing the strength of the nucleophile because it is mainly determined by the strength of the C^α^–Y bond, which remains constant. Therefore, the strain energy is not responsible for the reduced reactivity of the nucleophilic substitution reactions.

A weaker nucleophile, i.e., going from F^–^ to the weaker Lewis base Cl^–^, results in a weakening of the acid–base-like HOMO_X^–^_–LUMO_substrate_ interaction with the substrate over the entire course of the reaction. This loss of HOMO–LUMO interaction originates from the fact that the halide X^–^ n*p* atomic orbital (AO) decreases in energy from F^–^ to I^–^, which, consequently, increases the corresponding HOMO_X^–^_–LUMO_substrate_ orbital energy gap (Figure 3; see ref (14) for a detailed discussion of the difference in the AO orbital energies of halogen atoms and halide anions).

In contrast, changing the leaving-group from Y = F to Cl promotes both the S_N_2 and S_N_2′ reactivities by reducing the destabilizing strain energy (Figure 2b,d). The interaction energy, however, does not change upon altering the leaving-group and hence is not responsible for the observed enhanced reactivity. The less destabilizing strain originates directly from the weaker carbon–leaving-group bond descending in Group 17 (i.e., halogens), rendering it easier to break, thus requiring less energy.^15^ Notably, we recently found that when going down Group 17, the carbon–halogen bond, i.e., carbon–leaving-group bond, does not become weaker because of the decreasing electronegativity difference but, instead, due to the increasing steric (Pauli) repulsion across the C–Y bond for the larger halogen atoms.^16^

To analyze the competition between S_N_2 and S_N_2′, we collect in Figure 4 four panels showing the aliphatic S_N_2 and allylic S_N_2′ pathways of the model reactions: (a) F^–^ + H_2_C=CHCH_2_F, (b) F^–^ + H_2_C=CHCH_2_Cl, (c) Cl^–^ + H_2_C=CHCH_2_F, and (d) Cl^–^ + H_2_C=CHCH_2_Cl. The nature of the nucleophile changes in the vertical direction, whereas the leaving-group is altered in the horizontal direction. Several characteristic trends for the S_N_2 (attack at the σ*) and S_N_2′ (attack at the π*) pathways can be derived from the computed activation strain diagrams (ASDs). For all studied reactions, the aliphatic S_N_2 reaction pathway goes with a lower strain energy than the allylic S_N_2′ analog (see also Figure S1). This difference is the direct result of the required allylic rearrangement that occurs only along the S_N_2′ pathway. During both reactions, the C^α^–Y bond is being broken; however, for the S_N_2′ pathway, there is a concurrent reorganization of the allylic backbone, going from H_2_C^γ^=C^β^HC^α^H_2_···Y to H_2_C^γ^C^β^H=C^α^H_2_···Y. Resultingly, the “characteristic distortivity” along the S_N_2 pathway is inherently lower than along the corresponding S_N_2′ pathway. Notably, we have observed a similar relationship between structural deformation and reaction pathway when studying the S_N_2/E2 competition,^11^ where the S_N_2 pathway (one bond-breaking event in the substrate) goes with intrinsically less structural deformation, i.e., characteristic distortivity, than the E2 pathway (two bond-breaking events in the substrate). Importantly, the strain curves at the end of the nucleophilic substitution reactions, thus close to the product complex, begin to converge to nearly the same energy. This is because the product complexes of both the S_N_2 and S_N_2′ pathways are structurally similar.

A same product molecule is formed and only the site at which the leaving-group coordinates is different (see product complexes in Figure 1). This phenomenon of converging strain curves in the product complex contrasts with the S_N_2/E2 competition, in which the E2 reaction features, along the entire reaction pathway, an increasingly more destabilizing strain energy, as it forms a PC composed of entirely different product molecules than the S_N_2 reaction.^11^

**Figure 6:**
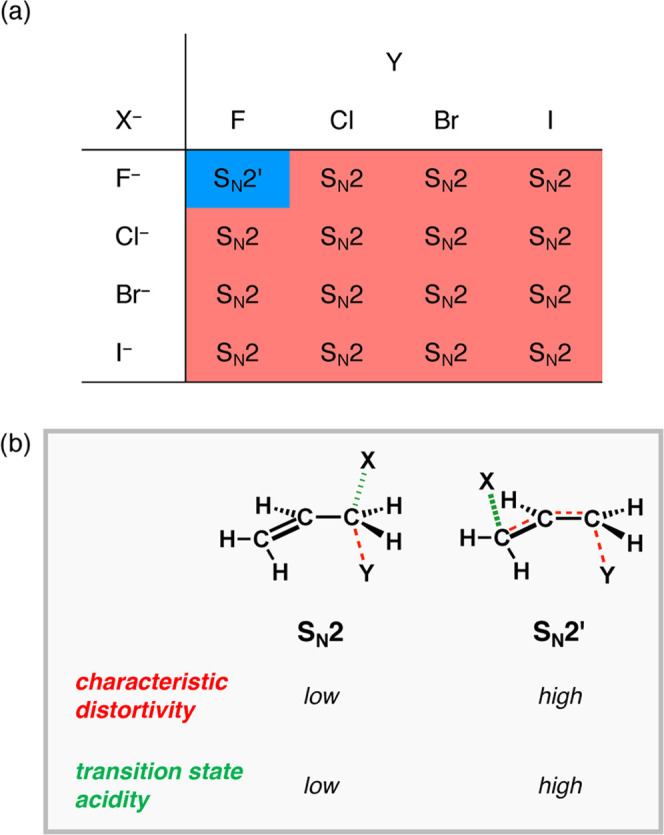
Schematic summary of the S_N_2/S_N_2′ competition. (a) Reaction pathway preference for X^–^ and H_2_C=CHCH_2_Y with X, Y = F, Cl, Br, and I. (b) “Characteristic distortivity” (destabilizing) and “transition state acidity” (stabilizing) of the aliphatic S_N_2 and allylic S_N_2′ reaction pathways, where the characteristic distortivity (deformation; red) is always higher along the S_N_2′ pathway than the S_N_2. However, this can be overcome using strong Lewis bases (nucleophiles), by the corresponding transition state acidity (lower-lying LUMO; green).

At the same time, the characteristic distortivity also has a profound effect on the electronic structure of the substrate (Figures 5 and 6b). In our reaction systems, a higher characteristic distortivity, i.e., more deformation of the substrate, gives rise to a lower-energy LUMO in the substrate. The reason for this is that the LUMO of the substrate has destabilizing antibonding character in the C^α^–Y and C^γ^=C^β^ bonds and stabilizing bonding character in the C^β^–C^α^ bond (Figure 5). The elongation of the C^α^–Y bond found during both the S_N_2 and S_N_2′ reactions reduces the antibonding overlap in this bond, resulting in a stabilization of the LUMO orbital energy. Additionally, the allylic rearrangement along the S_N_2′ pathway induces a stretch of the C^γ^=C^β^ bond and a contraction of the C^β^–C^α^, which results in less destabilizing antibonding character in the C^γ^=C^β^ bond and more bonding character in the C^β^–C^α^ bond. Both geometrical deformations lead to an additional stabilization of the LUMO of the substrate during the S_N_2′ reaction. Note that at the beginning of the reaction almost no deformation has taken place, and hence the LUMO energy difference between the S_N_2 and S_N_2′ is very small (Figure S2). Moreover, as previously discussed, the product complexes of both the S_N_2 and S_N_2′ pathways are comparable in structure, which causes the LUMO energies of the different reaction pathways to converge at the end of the reaction. This will, as discussed below, have direct implications on the observed reactivity trends when the transition states appear to be very early or late on the reaction coordinate.

Altogether, these findings allow us to translate the interaction energy emerging from our activation strain analyses, in analogy with our previous work on the S_N_2/E2 competition,^11^ into fundamental concepts that are intrinsically based on the strength of the Lewis acid (substrate) and Lewis base (nucleophile). It is well-known from acid–base chemistry that a more basic Lewis base (high-energy HOMO) will interact more strongly with a more acidic substrate (low-energy LUMO).^17^ We, therefore, use the, previously introduced by us,^11^ concept of “transition state acidity”, that is, the effective acidity of a deformed substrate in the transition state. As discussed earlier, the S_N_2 pathway goes with a less acidic substrate (higher-energy LUMO) than the S_N_2′ pathway (lower-energy LUMO). Therefore, the S_N_2′ pathway can dominate the S_N_2 pathway in the limit of a very strong interaction (stronger Lewis base), which we have observed for the reactions in which X, Y = F (Figures 4a and 6a). Changing the nucleophile from X^–^ = F^–^ to Cl^–^ has a profound effect on the preferred reaction pathway, shifting the preference from S_N_2′ for F^–^ (Figure 4a) to S_N_2 for Cl^–^ (Figure 4c). This is a direct consequence of the less stabilizing HOMO–LUMO interaction between nucleophile and substrate as we go from the stronger base F^–^ to the weaker base Cl^–^. The weaker interaction in the case of the latter is no longer able to overcome the higher strain associated with the higher characteristic distortivity along the S_N_2′ pathway and hence results in a preference for the S_N_2 pathway (Figure 6b). Likewise, for Y = Cl as leaving-group, going from X = F^–^ (Figure 4b) to X = Cl^–^ (Figure 4d), the preference for S_N_2 increases (ΔΔ*E*^‡^ = −2.0 to −5.7 kcal mol^–1^ for S_N_2 relative to *anti*-S_N_2′); however, since X = F^–^ follows already the S_N_2 pathway, no switch in mechanism is observed. Note that, in the case of the weaker nucleophile Cl^–^, the position of the transition states shifts to a later point on the reaction coordinate because of the less stabilizing and more shallow interaction curve. The occurrence of the TS at a later stage of the reaction causes a smaller difference in LUMO energy between the aliphatic S_N_2 and allylic S_N_2′ pathways and hence a smaller difference in “transition state acidity” (see also Figure S2). On the other hand, increasing the leaving-group ability, from Y = F to Cl (Figure 4a–d), shifts the position of the transition states to an early position on the reaction coordinate, especially for the S_N_2′ pathway. At these early stages of the reaction, almost no substrate deformation has taken place and hence no stabilization of the LUMO. This, ultimately, yields no apparent difference in “transition state acidity” between the pathways, and thus, we observe erosion of the S_N_2′ preference shifting to the less distortive S_N_2 pathway.

Our model can also explain the effect of solvation on the S_N_2 versus S_N_2′ competition. The localized charge density on the anionic nucleophile (Lewis base) is highly stabilized upon solvation, which leads to a stabilization of the HOMO of X^–^ (weaker nucleophile). Thus, the HOMO–LUMO interaction (acid–base-like interaction) between the Lewis base and substrate is weakened by solvation.^11c^ The weaker interaction ultimately causes a shift to the less distortive S_N_2 pathway. To showcase this fundamental effect, we have computed the S_N_2 and *anti-*S_N_2′ activation barriers of all our systems with the inclusion of solvation simulated with COSMO (dichloromethane). As expected, as a result of solvation, all reaction barriers increase significantly and follow the less distortive S_N_2 pathway (Table S2), which is a direct consequence of the weakening of the Lewis-basic nucleophiles. For example, by going from the gas phase to solution, the X, Y = F system switches from S_N_2′ to S_N_2. Note that these effects will be even more pronounced when the polarity of the solvent increases.^11c^

Lastly, we investigated the S_N_2/S_N_2′ competition of more commonly used nucleophiles MeO^–^, MeS^–^, MeSe^–^, and MeTe^–^ with C_2_H_5_F as a substrate to test our model (Table S3). As discussed earlier, strong nucleophiles will have a more favorable interaction with the substrate than weak nucleophiles and, therefore, the former will be able to overcome the characteristic high distortivity accompanied with the S_N_2′ reaction. Hence, based on the strength of the nucleophile, which is associated with the energy of the HOMO, one can anticipate the preferred reaction pathway. By going down a group in the periodic table, the HOMO energy becomes stabilized, resulting in a lower nucleophilicity along MeO^–^, MeS^–^, MeSe^–^, and MeTe^–^. As expected, the S_N_2′ preference erodes on going from MeO^–^ to MeS^–^, MeSe^–^, and MeTe^–^ (ΔΔ*E*^‡^ = +9.1, +6.1, +2.9, +1.6 kcal mol^–1^ for S_N_2 relative to *anti*-S_N_2′). The weakest nucleophile in this series still has a preference for the S_N_2′ pathway as a result of the intrinsic higher reactivity of Group 16 (chalcogens) compared to Group 17 (halogens) in the periodic table.

## Conclusions

Bimolecular nucleophilic substitution at archetypal allylic substrates X^–^ + H_2_C=CHCH_2_Y (X, Y = F, Cl, Br, I) can follow two distinct mechanistic pathways: aliphatic S_N_2 or allylic S_N_2′ substitution. The aliphatic S_N_2 pathway is in general favored over the allylic S_N_2′ mechanism. For our studied systems, we found that the latter only dominates in the case of a strong nucleophile (X^–^ = F^–^) in combination with a poor leaving-group (Y = F). Both mechanistic pathways are accelerated as the Lewis basicity of the anionic nucleophile increases, along X^–^ = I^–^ to F^–^, and the carbon–leaving-group (C–Y) bond becomes weaker, along Y = F to I. These and other insights emerge from our detailed quantum chemical analyses based on relativistic DFT.

Our activation strain analyses reveal that a stronger, more Lewis-basic nucleophile X^–^ (e.g., going from Cl^–^ to F^–^) lowers the activation energy for both the S_N_2 and S_N_2′ pathways, because of a more stabilizing acid–base-like HOMO_X^–^_–LUMO_substrate_ interaction with the substrate over the entire course of the reaction. A leaving-group Y possessing a weaker C–Y bond in the substrate (e.g., going from C–F to C–Cl) also lowers the activation energy for both the S_N_2 and S_N_2′ pathways, because a less destabilizing activation strain needs to be overcome to break such a weaker carbon–leaving-group bond.

In analogy to the previously studied S_N_2/E2 competition,^11^ the propensity of the Lewis-basic nucleophile to follow an S_N_2 or S_N_2′ pathway is found to be steered by the distinct structural deformation of the substrate during the course of the reaction in combination with the nature of the Lewis base and the leaving-group. The allylic S_N_2′ pathway (C–Y bond breaking and allylic rearrangement in the substrate) is characterized by a higher “characteristic distortivity” than the aliphatic S_N_2 pathway (only C–Y bond breaking in the substrate). The higher characteristic distortivity of the S_N_2′ pathway is associated with a higher activation strain, which contributes to the S_N_2′ activation energy being higher than the S_N_2 one.

But the higher distortivity of the S_N_2′ pathway also stabilizes the LUMO (lower-energy LUMO) of the substrate and furnishes a substrate with an effectively higher TS acidity. In the case of strong Lewis-basic nucleophiles (X^–^ = F^–^ in our case), this can lead to sufficiently stabilizing HOMO–LUMO interaction between nucleophile and substrate that can overcome the higher activation strain of the S_N_2′ pathway, making it the dominant mechanism over S_N_2. For the weaker Lewis bases (X^–^ = Cl^–^, Br^–^, I^–^), and thus also for (strongly) solvated Lewis bases, the HOMO–LUMO interaction is not strong enough to overcome the higher activation strain of the allylic S_N_2′ pathway and, thus, the less distortive aliphatic S_N_2 substitution emerges as the dominant pathway. Our present work demonstrates the more general applicability of the concepts of “characteristic distortivity” and “transition state acidity” to not only explain but also predict reactivity trends of fundamental organic reactions.

## Computational Methods

### Computational Details

All density functional theory (DFT) calculations were performed using the Amsterdam Density Functional (ADF2018.105) software package.^18^ The generalized gradient approximation (GGA) exchange–correlation functional OLYP was used for all geometry optimizations, which consists of the optimized exchange (OPTX) functional proposed by Handy and co-workers^19a^ and the Lee–Yang–Parr (LYP) correlation functional.^19b^ Our benchmark studies have shown that OLYP gives accurate nucleophilic substitution stationary point geometries.^20^ Scalar relativistic effects are accounted for using the zeroth-order regular approximation (ZORA).^21^ The basis set used, denoted QZ4P, is of quadruple-ζ quality for all atoms and has been improved by four sets of polarization functions.^22^ This large basis set is required for small anionic species (e.g., F^–^). All solution-phase calculations used COSMO to simulate bulk solvation. For these calculations, the optimized stationary points in the gas phase were fully reoptimized at COSMO(DCM)-ZORA-OLYP/QZ4P.^23^ The accuracies of the fit scheme (Zlm fit) and the integration grid (Becke grid) were, for all calculations, set to VERYGOOD.^24^ No symmetry constraints were used for all computations. All calculated stationary points have been verified by performing a vibrational analysis calculation,^25^ to be energy minima (no imaginary frequencies) or transition states (only one imaginary frequency). The character of the normal mode associated with the imaginary frequency of the transition state has been inspected to ensure that it is associated with the reaction of interest. The stationary point energies have been refined by performing single points at ZORA-M06-2X^26^/QZ4P, using ADF,^18^ as well as (TightPNO)DLPNO-CCSD(T)/CBS(3,4/def2),^27^ using ORCA 5.0.1,^28^ on the ZORA-OLYP/QZ4P geometries. The potential energy surfaces of the studied bimolecular nucleophilic substitution reactions were obtained by means of intrinsic reaction coordinate (IRC) calculations, which confirmed the correct transition state of interest.^29^ The IRC calculations were analyzed using the PyFrag 2019 program.^30^ The optimized structures were illustrated using CYLview.^31^

### Activation Strain Model of Reactivity

The activation strain model (ASM) of chemical reactivity,^8^ also known as the distortion/interaction model,^32^ is a fragment-based approach in which the potential energy surface (PES) can be described with respect to, and understood in terms of the characteristics of, the reactants. It considers the rigidity of the reactants and to which extent they need to deform during the reaction, plus their capability to interact with each other as the reaction proceeds. With the help of this model, we decompose the gas-phase total energy, Δ*E*(ζ), into the strain and interaction energy, Δ*E*_strain_(ζ) and Δ*E*_int_(ζ), respectively, and project these values onto the reaction coordinate ζ (eq 1).
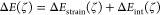
1

In this equation, the strain energy, Δ*E*_strain_(ζ), is the penalty that needs to be paid to deform the reactants from their equilibrium to the geometry they adopt during the reaction at the point ζ of the reaction coordinate. On the other hand, the interaction energy, Δ*E*_int_(ζ), accounts for all of the chemical interactions that occur between these two deformed reactants along the reaction coordinate. Note that at the position along the reaction coordinate where the destabilization of the strain terms increases with the same slope as the stabilization of the interaction energy term increases, i.e., dΔ*E*_strain_(ζ)/dζ = −dΔ*E*_int_(ζ)/dζ, the derivative of the total energy Δ*E*(ζ) with respect to the reaction coordinate is zero (dΔ*E*(ζ)/dζ = 0).^8^ At this point, the reaction profile reaches either a maximum (transition state) or a minimum (reactant complex or product complex).

In the herein presented activation strain and accompanied energy decomposition diagrams, the intrinsic reaction coordinate (IRC) is projected onto the carbon–leaving-group (C^α^···Y) distance. This critical reaction coordinate undergoes a well-defined change during the reaction from the reactant via the transition state to the product.^11,13^

## Supporting Information Available

Additional computational results and Cartesian coordinates, energies, and the number of imaginary frequencies of all stationary points (PDF)
