# Supplementary material for: SN2 versus SN2′ Competition
Source: J Org Chem. 2022 Jun 24;87(14):8892–901. doi: 10.1021/acs.joc.2c00527 (PMC9295157; doi:10.1021/acs.joc.2c00527)
Supplement: Supplementary file 1 — jo2c00527_si_001.pdf [file jo2c00527_si_001.pdf]

The S_N2 versus S_N2' Competition

Thomas Hansen,[+,†,‡,§] Pascal Vermeeren,[+,†] Lea de Jong,[+,†] F. Matthias Bickelhaupt,[†,§] and Trevor A. Hamlin* [†]

[†] Department of Theoretical Chemistry, Amsterdam Institute of Molecular and Life Sciences (AIMMS), Amsterdam Center for Multiscale Modeling (ACMM), Vrije Universiteit Amsterdam,
De Boelelaan 1083, 1081 HV Amsterdam, The Netherlands.
Email: f.m.bickelhaupt@vu.nl; t.a.hamlin@vu.nl
[‡] Leiden Institute of Chemistry, Leiden University, Einsteinweg 55,
2333 CC Leiden, The Netherlands.
[§] Departament de Química Inorgànica i Orgànica & IQTCUB, Universitat de Barcelona, 08028 Barcelona, Spain
[§] Institute for Molecules and Materials (IMM), Radboud University, Heyendaalseweg 135,
6525 AJ Nijmegen, The Netherlands.
[+] These authors contributed equally to this work

Contents

Figure S1. Activation-strain analysis (in kcal mol^{-1}) of S_N2 (blue) and *anti*-S_N2' (blue) reactions of X^- + $H_2C{=}CHCH_2Y$ (X, Y = F, Cl, Br, I) along the IRC projected onto the C•••Y stretch (in Å), computed at ZORA-OLYP/QZ4P. Dots indicate transition states. — S2

Quantitative insight into the electronic structure of the substrate — S3

Figure S2. Characteristic distortivity represented by substrate C^{β}–C^{α} and C^{γ}=C^{β} distances and the substrate LUMO energy of S_N2 and S_N2' reactions X^- + $H_2C{=}CHCH_2Y$ (X, Y = F, Cl) along the IRC projected onto the C^{α}•••Cl bond stretch. The transition states are indicated by a vertical line. Computed at ZORA-M06-2X/QZ4P//ZORA-OLYP/QZ4P. — S4

Table S1. Energies relative to the reactants for all stationary points of the S_N2, *anti*-S_N2', and *syn*-S_N2' pathways following X^- + $H_2C{=}CHCH_2Y$ → $H_2C{=}CHCH_2X$ + Y^- in kcal mol^{-1}. — S5

Table S2. Energies relative to the reactants for the transition states in solution of the S_N2 and *anti*-S_N2' pathways following X^- + $H_2C{=}CHCH_2Y$ → $H_2C{=}CHCH_2X$ + Y^- in kcal mol^{-1}. — S6

Table S3. Energies relative to the reactants for the transition states of the S_N2 and *anti*-S_N2' pathways following MeZ^- + $H_2C{=}CHCH_2Y$ → $H_2C{=}CHCH_2X$ + Y^- in kcal mol^{-1}. — S6

Table S4. Cartesian coordinates (in Å), energies (E, H, and G, in kcal mol^{-1}), and number of imaginary vibrational frequencies (N_{imag}) of all stationary points in the gas phase, computed at ZORA-OLYP/QZ4P. Not all reactions are shown because many stationary points are shared over two reaction systems due to symmetry (*e.g.*, reaction 1b = 2a). — S7

Table S5. Cartesian coordinates (in Å), energies (E, H, and G, in kcal mol^{-1}), and number of imaginary vibrational frequencies (N_{imag}) of all stationary points in solution, computed at COSMO(DCM)-ZORA-OLYP/QZ4P. Not all reactions are shown because many stationary points are shared over two reaction systems due to symmetry (*e.g.*, reaction 1b = 2a). — S30

Table S6. Cartesian coordinates (in Å), energies (E, H, and G, in kcal mol^{-1}), and number of imaginary vibrational frequencies (N_{imag}) of all stationary points for MeZ^- + $H_2C{=}CHCH_2Y$ systems, computed at ZORA-OLYP/QZ4P. — S39

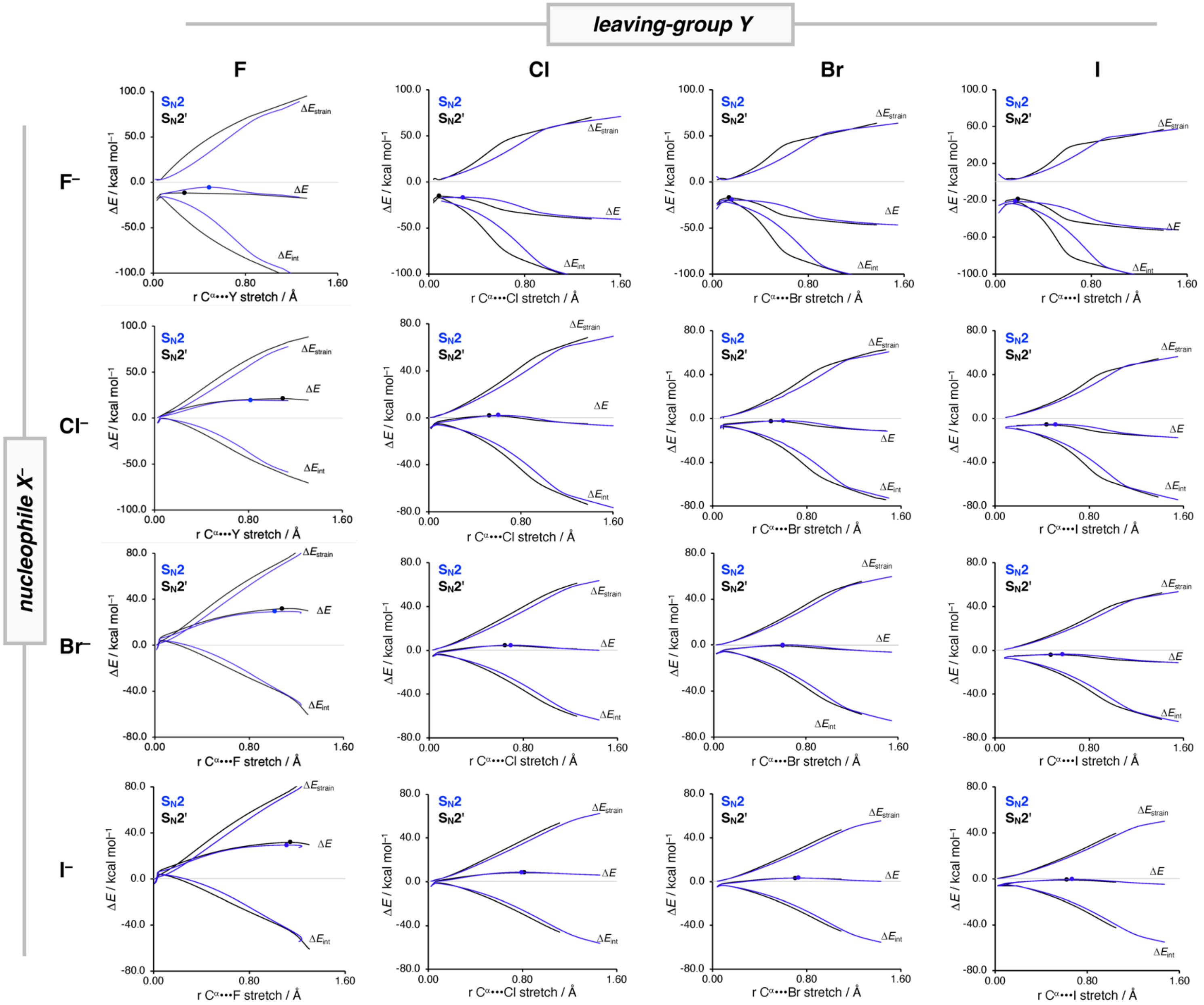

Figure S1. Activation-strain analysis (in kcal mol^{-1}) of S_N2 (blue) and *anti*-S_N2' (blue) reactions of X^- + H_2C=$CHCH_2Y$ (X, Y = F, Cl, Br, I) along the IRC projected onto the C•••Y stretch (in Å), computed at ZORA-OLYP/QZ4P. Dots indicate transition states.

Quantitative insight into the electronic structure of the substrate

To get quantitative insight into the effect of the characteristic distortivity on the electronic structure of the substrate for each pathway, we depict in Figure S2 the deformation of the backbone of the substrate, represented by the C^{β}–C^{α} and C^{γ}=C^{β} distances as well as the corresponding LUMO energy along the reaction coordinate of representative reactions of X^{-} + H_2C=$CHCH_2Y$ (X, Y = F, Cl) shown in Figure 4. As already discussed, both pathways have a distinct deformation of the backbone of the substrate along the reaction pathways. For the S_N2', an allylic rearrangement takes place (Figure S1; blue), and in all cases, the contraction of the C^{β}–C^{α} bond and elongation of the C^{γ}=C^{β} bond of the substrate during this rearrangement occur simultaneously. In contrast, for the aliphatic S_N2 pathway, only a minor contraction of the C^{β}–C^{α} happens along the reaction (Figure S1; red). This results, for all reaction systems, in a more stabilized substrate LUMO for the S_N2' pathway than the S_N2 along the reaction coordinate. The reason for this is that the LUMO of the substrate has destabilizing antibonding character in the C^{α}–Y and C^{γ}=C^{β} bonds and stabilizing bonding character in the C^{β}–C^{α} bond (Figure 5). The elongation of the C^{α}–Y bond found during both the aliphatic S_N2 and allylic S_N2' reaction reduces the antibonding overlap in this bond, resulting in a stabilization of the LUMO orbital energy. Additionally, the allylic rearrangement along the S_N2' pathway induces a stretch of the C^{γ}=C^{β} bond and a contraction of the C^{β}–C^{α}, which results in less destabilizing antibonding character in the C^{γ}=C^{β} bond and more bonding character in the C^{β}–C^{α} bond. Both lead to an additional stabilization of the LUMO of the substrate during the S_N2' reaction.

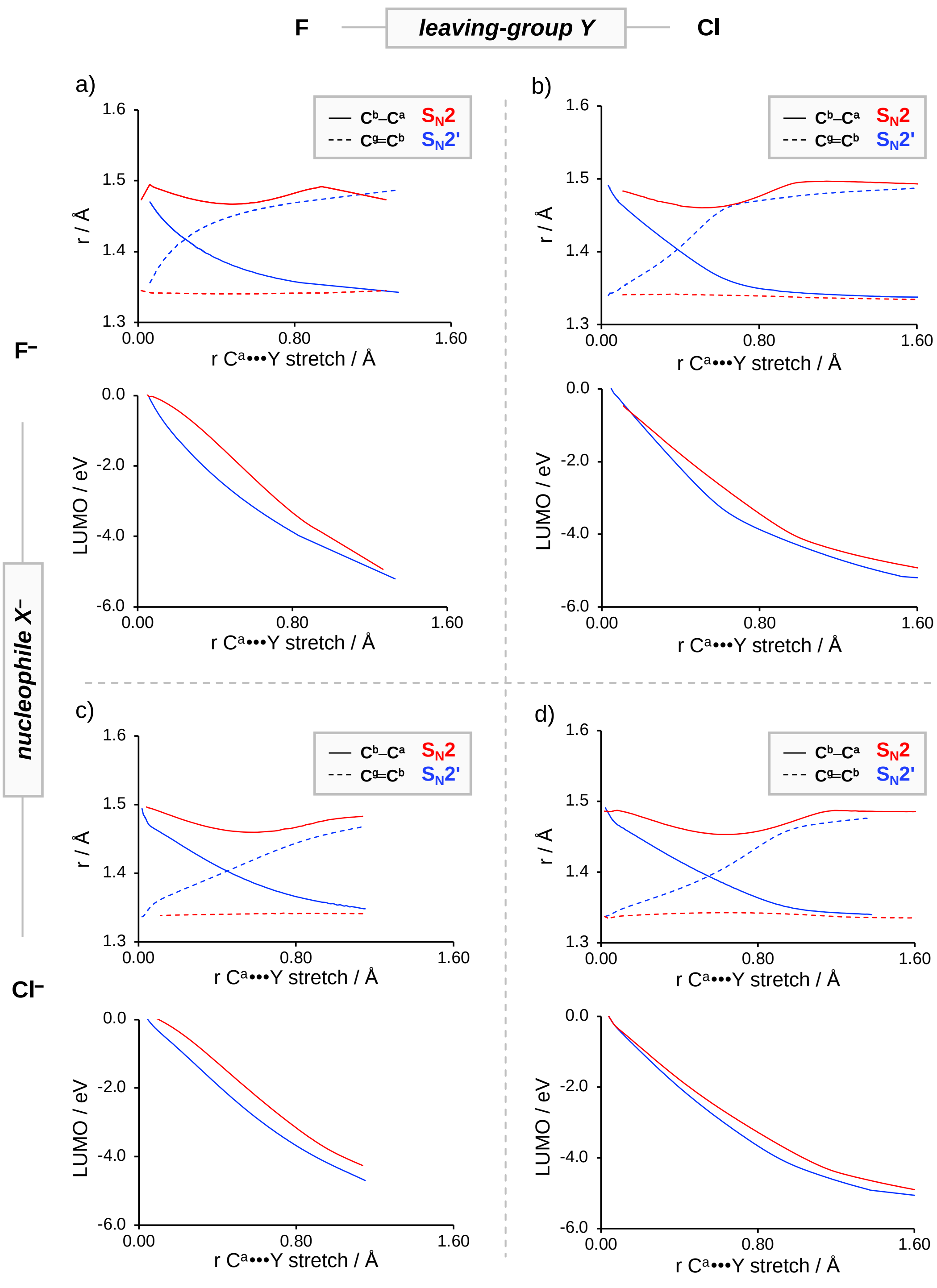

Figure S2. Characteristic distortivity represented by substrate C^{β}–C^{α} and C^{γ}=C^{β} distances and the substrate LUMO energy of aliphatic S_N2 and allylic S_N2' reactions X^{-} + H_2C=$CHCH_2Y$ (X, Y = F, Cl) along the IRC projected onto the C^{α}•••Cl bond stretch. The transition states are indicated by a vertical line. Computed at ZORA-M06-2X/QZ4P//ZORA-OLYP/QZ4P.

Table S1. Energies relative to the reactants for all stationary points of the S_N2, *anti*-S_N2', and *syn*-S_N2' pathways following $X^- + H_2C{=}CHCH_2Y \rightarrow H_2C{=}CHCH_2X + Y^-$ in kcal mol^{-1}.[a]

		Y			
**X⁻**	**Species**	**F (a)**	**Cl (b)**	**Br (c)**	**I (d)**
**F⁻ (1)**	RC-S_N2	−20.3	−23.0	−23.8	−25.4
	RC-S_N2'	−19.6	−22.3	−23.0	−24.2
	TS-S_N2	−5.6	−17.1	−19.5	−21.2
	TS-a-S_N2'	−11.8	−15.4	−17.1	−18.7
	TS-s-S_N2'	−11.1	−13.8	−15.8	−17.7
	PC-S_N2	−20.3	−43.0	−48.7	−54.5
	PC-S_N2'	−19.6	−44.2	−49.8	−55.4
	P	0.0	−36.6	−43.3	−50.5
**Cl⁻ (2)**	RC-S_N2	−6.4	−8.1	−8.5	−9.1
	RC-S_N2'	−7.6	−8.8	−9.1	−9.4
	TS-S_N2	19.5	2.3	−2.2	−5.4
	TS-a-S_N2'	21.1	1.9	−2.6	−5.7
	TS-s-S_N2'	22.8	4.7	0.3	−2.8
	PC-S_N2	13.6	−8.1	−13.6	−19.0
	PC-S_N2'	14.3	−8.8	−14.2	−19.6
	P	36.6	0.0	−7.6	−14.0
**Br⁻ (3)**	RC-S_N2	−5.3	−6.8	−7.1	−7.7
	RC-S_N2'	−6.4	−7.4	−7.6	−8.0
	TS-S_N2	23.9	4.6	−0.1	−3.6
	TS-a-S_N2'	26.3	4.2	−0.6	−4.0
	TS-s-S_N2'	27.6	7.2	2.4	−1.1
	PC-S_N2	19.6	−1.6	−7.1	−12.5
	PC-S_N2'	20.4	−2.2	−7.6	−12.9
	P	43.4	6.8	0.0	−7.1
**I⁻ (4)**	RC-S_N2	−4.0	−5.1	−5.4	−5.9
	RC-S_N2'	−4.9	−5.6	−5.8	−6.0
	TS-S_N2	29.3	8.5	3.5	−0.4
	TS-a-S_N2'	31.8	8.2	3.1	−0.9
	TS-s-S_N2'	32.8	11.2	6.0	2.1
	PC-S_N2	25.1	4.8	−0.6	−5.9
	PC-S_N2'	26.3	4.5	−0.9	−6.0
	P	50.5	13.9	7.1	0.0

[a] Computed at ZORA-OLYP/QZ4P.

Table S2. Energies relative to the reactants for the transition states in solution of the S_N2 and *anti*-S_N2' pathways following X^- + H_2C=$CHCH_2Y$ → H_2C=$CHCH_2X$ + Y^- in kcal mol^{-1}.[a]

		Y			
**X⁻**	**Species**	**F (a)**	**Cl (b)**	**Br (c)**	**I (d)**
**F^- (1)**	TS-S_N2	28.4	18.6	7.1	2.0
	TS-*anti*-S_N2'	30.9	23.6	20.4	20.0
**Cl^- (2)**	TS-S_N2	33.4	21.6	21.5	17.5
	TS-*anti*-S_N2'	38.5	26.1	22.4	21.3
**Br^- (3)**	TS-S_N2	25.1	21.5	17.8	17.1
	TS-*anti*-S_N2'	38.4	25.6	21.6	21.0
**I^- (4)**	TS-S_N2	20.2	20.9	17.3	16.4
	TS-*anti*-S_N2'	38.2	24.7	21.0	20.0

[a] Computed at COSMO(DCM)-ZORA-M06-2X/QZ4P//COSMO(DCM)-ZORA-OLYP/QZ4P.

Table S3. Energies relative to the reactants for the transition states of the S_N2 and *anti*-S_N2' pathways following MeZ^- + H_2C=$CHCH_2Y$ → H_2C=$CHCH_2X$ + Y^- in kcal mol^{-1}.[a]

**MeZ^-**		TS ($\Delta E^{\ddagger}$)
**MeO^-**	TS-S_N2	1.3
	TS-*anti*-S_N2'	–7.9
**MeS^-**	TS-S_N2	12.6
	TS-*anti*-S_N2'	6.5
**$MeSe^-$**	TS-S_N2	16.5
	TS-*anti*-S_N2'	13.6
**$MeTe^-$**	TS-S_N2	22.4
	TS-*anti*-S_N2'	20.8

[a] Computed at ZORA-M06-2X/QZ4P//ZORA-OLYP/QZ4P.

Table S4. Cartesian coordinates (in Å), energies (E, H, and G, in kcal mol^{-1}), and number of imaginary vibrational frequencies (N_{imag}) of all stationary points in the gas phase, computed at ZORA-OLYP/QZ4P. Not all reactions are shown because many stationary points are shared over two reaction systems due to symmetry (*e.g.*, reaction 1b = 2a).

3-fluoroprop-1-ene:

E = −1089.90
H = −1041.77
G = −1062.27
N_{imag} = 0

C	0.37758600	-0.76582700	2.13118000
C	-0.26522900	-0.39669100	1.02105200
C	0.29234500	0.53891400	-0.00271400
H	-0.32219300	1.44567400	-0.08770400
H	-0.08363200	-1.42227300	2.86367300
H	1.38387900	-0.41720700	2.35250200
H	-1.27146500	-0.76552800	0.82336100
H	1.32474900	0.82540300	0.22695200
F	0.29012300	-0.06426900	-1.27710200

3-chloroprop-1-ene:

E = −1049.86
H = −1002.39
G = −1023.64
N_{imag} = 0

C	0.44533600	-0.64511200	2.19310800
C	-0.30908500	-0.36970900	1.12587300
C	0.17170800	0.39272600	-0.06160900
Cl	0.09833500	-0.60570800	-1.57778800
H	0.04088100	-1.17839900	3.04854500
H	1.48885800	-0.34452900	2.25154500
H	-1.34836000	-0.69361300	1.09553500
H	1.20876900	0.71334700	0.04652500
H	-0.45776800	1.26258100	-0.26650200

3-bromoprop-1-ene:

E = −1036.32
H = −989.10
G = −1011.16
N_{imag} = 0

Br	0.26840400	-0.47420400	-1.74223300
C	0.38642800	-0.74101800	2.14559100
C	-0.31790500	-0.34048800	1.08200500
C	0.23096900	0.50306800	-0.00899200
H	-0.39235500	1.37309700	-0.22125400
H	-0.06895600	-1.33359300	2.93365200
H	1.43719400	-0.48826000	2.26638100
H	-1.36613200	-0.62092200	0.99035400
H	1.26040000	0.81236900	0.16849200

3-iodoprop-1-ene:

E = -1023.83
H = -976.84
G = -999.49
N_{imag} = 0

C	0.44024200	-0.81021700	2.09242100
C	-0.31025300	-0.34427200	1.08705700
C	0.18291800	0.57090500	0.03279400
H	-0.47561400	1.42366400	-0.13358100
H	0.01915200	-1.45520300	2.85792100
H	1.49496100	-0.56205200	2.18471100
H	-1.36047400	-0.62603800	1.02618100
H	1.20634000	0.90614000	0.19412900
I	0.22083200	-0.38843500	-1.94821400

Fluoride:

E = -91.22
H = -90.33
G = -98.08
N_{imag} = 0

F	0.00000000	0.00000000	0.00000000

Chloride:

E = -87.76
H = -86.87
G = -94.62
N_{imag} = 0

Cl	0.00000000	0.00000000	0.00000000

Bromide:

E = -81.03
H = -80.14
G = -87.89
N_{imag} = 0

Br	0.00000000	0.00000000	0.00000000

Iodide:

E = -75.64
H = -74.75
G = -82.50
N_{imag} = 0

I	0.00000000	0.00000000	0.00000000

1a-RC/PC-S_N2:

E = -1200.03
H = -1151.82
G = -1175.85
***N*imag** = 0

C	0.73817200	0.08311000	-0.68046600
F	-0.70063500	-1.81971900	0.56555700

F	0.37852000	0.10227500	-2.07393300
C	0.55083500	1.43107200	-0.10856900
C	1.36781900	2.06573700	0.75106900
H	0.07487700	-0.71811100	-0.14194200
H	1.78821400	-0.23882700	-0.64148300
H	-0.40289500	1.89983300	-0.36262700
H	1.07985000	3.00030500	1.22833100
H	2.32433800	1.64008400	1.04943700

1a-RC/PC-S_N2′:
E = -1201.41
H = -1153.32
G = -1177.12
***N*imag** = 0

C	-0.32849500	0.31837300	0.02962800
C	0.11010900	1.58455400	0.04864300
C	1.57230100	1.88872700	0.00779300
F	2.02182700	-1.30105600	-0.07901700
F	1.85600600	2.87151100	-1.00400900
H	0.42029000	-0.50833500	-0.03246700
H	-1.39963800	0.11401500	0.10318700
H	-0.56627000	2.44144100	0.12815100
H	1.92100300	2.34626700	0.94634500
H	2.14093600	0.97465800	-0.20476600

1a-TS-S_N2:
E = -1186.74
H = -1138.48
G = -1161.83
***N*imag** = 1,-414 cm^{-1}

C	0.61748800	-0.24570800	0.00065900
F	-1.27314000	-0.15225500	0.02438900
F	2.47454800	-0.61270700	0.01360700
C	0.78833200	1.13244800	0.47449400
C	0.91925600	2.22062100	-0.29673200
H	0.58877400	-0.45428600	-1.05570600
H	0.52003000	-1.05520400	0.69941200
H	0.80678500	1.25203700	1.55760500
H	1.04254100	3.21137800	0.13545800
H	0.90733500	2.15407400	-1.38216600

1a-TS-a-S_N2′:
E = - 1192.88
H = -1144.47
G = -1167.17
***N*imag** = 1, -135.14068 cm^{-1}

C	0.89078500	-0.17089700	0.05074000
C	0.77966400	1.14622600	0.56296700
C	0.66045100	2.29634700	-0.25732200
F	2.42834600	-0.73981400	-0.12679400

F	-0.87944400	2.80581400	-0.55134000
H	0.56278000	-0.30388400	-0.98280900
H	0.54071200	-0.98042000	0.69676400
H	0.78629300	1.28193200	1.64503800
H	1.01732900	3.23965600	0.16465700
H	0.97620400	2.16942100	-1.29544900

1a-TS-s-S_N2′:
E = -1192.23
H = -1143.66
G = -1166.20
***N*imag** = 1, -68.23174 cm^{-1}

C	-0.17529565	0.30027387	-0.04101325
C	0.51343867	1.42951515	0.36188143
C	1.71050484	1.92566050	-0.29954352
F	-1.59702636	0.46243301	-1.29694778
F	1.51162972	3.07277795	-1.24701770
H	0.32152478	-0.39956546	-0.70916720
H	-0.89191696	-0.14818214	0.63990714
H	0.09536588	2.04825937	1.15748962
H	2.45011974	2.35264625	0.39285025
H	2.18324263	1.16055410	-0.92713428

2b-RC/PC-S_N2:
E = -1145.68
H = -1096.75
G = -1123.43
***N*imag** = 0

C	1.15583600	0.06951800	-0.32378800
C	1.21579200	1.38749800	0.36069700
C	1.35133800	2.56518300	-0.25828900
Cl	2.58017800	-0.97388600	0.17556300
Cl	-2.10754200	-1.14063800	0.24212100
H	1.22873300	0.16118500	-1.40853200
H	0.24301500	-0.48959800	-0.05544100
H	1.08744600	1.35905400	1.44209200
H	1.32382300	3.50261700	0.29207300
H	1.46418600	2.63329400	-1.33836000

2b-RC/PC-S_N2′:
E = -1146.42
H = -1097.33
G = -1123.85
***N*imag** = 0

C	1.00973500	-0.03313900	-0.14181800
C	1.52684800	1.05270400	0.73930900
C	1.16794000	2.33314000	0.59499000
Cl	2.38910800	-1.00848100	-0.86518100
Cl	-1.49023700	1.95746700	-2.12094700
H	0.40868000	0.35237900	-0.97052300

H	0.43374200	-0.77501400	0.41838800
H	2.19831400	0.74956400	1.54429500
H	1.53235200	3.09038600	1.28756700
H	0.48288500	2.63875900	-0.19492300

2b-TS-S$_N$2:
E = -1135.28
H = -1087.29
G = -1112.51
***N*imag** = 1, -263 cm^{-1}

C	0.62442100	-0.18946500	0.00297800
C	0.79249400	1.17066700	0.48585400
C	0.92415700	2.25799500	-0.29112500
Cl	2.98553200	-0.85564000	-0.00187100
Cl	-1.82736200	-0.26465200	0.00810800
H	0.59650700	-0.39996100	-1.05266200
H	0.52530500	-1.00911600	0.68751000
H	0.80937600	1.28828800	1.56788400
H	1.04670800	3.24799500	0.14002600
H	0.91318700	2.18819100	-1.37567700

2b-TS-a-S$_N$2′:
E = -1135.73
H = -1087.61
G = -1161.83
***N*imag** = 1, -176 cm^{-1}

C	0.81080500	-0.15471400	0.06313100
C	0.78443400	1.14664300	0.56334500
C	0.73956800	2.28362300	-0.24281600
Cl	2.97442200	-1.06987000	-0.20207100
Cl	-1.43366200	3.10988800	-0.66814800
H	0.60121100	-0.32248800	-0.98723400
H	0.59886300	-0.99081000	0.71727700
H	0.79937000	1.28264000	1.64380000
H	0.96609900	3.25500600	0.17791700
H	0.92028800	2.18534500	-1.30725100

2b-TS-s-S$_N$2′:
E = -1132.88
H = -1084.82
G = -1109.53
***N*imag** = 1, -174 cm^{-1}

C	-0.20960200	0.27923300	-0.08157300
C	0.48136400	1.42812200	0.32175700
C	1.70298200	1.84007000	-0.22415300
Cl	-2.05493400	0.52209500	-1.43316500
Cl	1.70782800	3.59234200	-1.71462200
H	0.26997700	-0.39264100	-0.78604500
H	-0.87049300	-0.20003000	0.63021100
H	0.04797000	2.03193700	1.11808000

H	2.36417300	2.44008400	0.38898500
H	2.20206300	1.18389000	-0.92988100

3c-RC/PC-S_N2:
E = -1124.96
H = -1076.08
G = -1104.43
***N*imag** = 0

C	1.07738000	-0.22368900	0.39344500
C	1.34509900	1.23319200	0.48148800
C	0.64677000	2.14413100	-0.20735600
Br	2.73251500	-1.22239900	-0.16556800
Br	-2.35310300	-0.18620900	-1.62890700
H	0.30533200	-0.48053300	-0.33476900
H	0.85462500	-0.67362900	1.36262100
H	2.12895500	1.54605900	1.17236700
H	0.84452400	3.20657100	-0.07697400
H	-0.15595100	1.85072300	-0.88098100

3c-RC/PC-S_N2′:
E = -1124.96
H = -1076.09
G = -1104.44
***N*imag** = 0

C	0.93148000	1.53772900	0.96519200
C	1.93753000	1.15785800	-0.05727900
C	1.64092900	0.40858100	-1.12621700
Br	1.50759600	0.93104600	2.79539100
Br	-2.40562300	0.36931700	-0.86499500
H	-0.05176100	1.09514300	0.79283100
H	0.84766200	2.61794400	1.09744100
H	2.94625300	1.54975000	0.07892200
H	2.39867000	0.19229100	-1.87717800
H	0.63351200	0.03136300	-1.29065400

3c-TS-S_N2:
E = -1135.28
H = -1087.29
G = -1112.51
***N*imag** = 1, −263 cm^{-1}

C	0.62623100	-0.17515200	0.00344000
C	0.79298100	1.18077100	0.48515600
C	0.92628500	2.27105600	-0.29087800
Br	3.13774400	-0.94127200	0.00184700
Br	-1.99586600	-0.30980400	0.00214100
H	0.59995700	-0.38839600	-1.05247400
H	0.52553000	-0.99586900	0.68759800
H	0.80820200	1.29961200	1.56712900
H	1.04791300	3.25988400	0.14256500
H	0.91729400	2.20332800	-1.37548700

3c-TS-a-S_N2′:
E = -1117.94
H = -1070.00
G = -1096.65
***N*imag** = 1, −125 cm^{-1}

C	0.69008500	-0.15224200	0.06995000
C	0.78592800	1.14671300	0.56269900
C	0.85966600	2.28059300	-0.24228000
Br	2.92679400	-1.36722900	-0.18103100
Br	-1.38789900	3.40945500	-0.71010200
H	0.49427900	-0.31335800	-0.98391800
H	0.41893700	-0.96735200	0.72790000
H	0.80525500	1.28398400	1.64301900
H	1.15104300	3.23284100	0.18088800
H	1.01730800	2.17185700	-1.30917500

3c-TS-s-S_N2′:
E = -1132.88
H = -1084.82
G = -1109.53
***N*imag** = 1, −174 cm^{-1}

C	-0.20313400	0.27379200	-0.07809000
C	0.48289200	1.42501300	0.31614100
C	1.70734100	1.83266500	-0.21865900
Br	-2.22901000	0.51384800	-1.52850200
Br	1.74388500	3.75463700	-1.82177900
H	0.25712800	-0.39659800	-0.79584600
H	-0.89047600	-0.18917200	0.61779100
H	0.04867900	2.02890800	1.11206700
H	2.35582300	2.45988300	0.37893500
H	2.20835200	1.19523300	-0.93915400

4d-RC/PC-S_N2:
E = -1105.33
H = -1056.72
G = -1086.72
***N*imag** = 0

C	1.28628800	0.02002800	-0.27846300
C	1.32085500	1.33887700	0.38496100
C	1.50585600	2.50623400	-0.24487600
I	3.12143100	-1.15577600	0.19521900
I	-2.55598800	-1.04477200	0.47388300
H	1.29675900	0.07351100	-1.36573700
H	0.47559000	-0.62082700	0.07546000
H	1.13924600	1.33292200	1.45840800
H	1.46996900	3.44990700	0.29295300
H	1.67389300	2.55767200	-1.31840200

4d-RC/PC-S$_N$2′:
E = -1105.46
H = -1057.36
G = -1085.12
***N*imag** = 0

C	0.24493400	0.13813000	0.94772100
C	0.12934400	1.45768300	0.74836800
C	0.61897200	2.14443600	-0.46740000
I	3.28688800	-0.76285800	-2.15936400
I	-1.04272000	3.15297400	-1.54651500
H	0.70645500	-0.51160200	0.20870100
H	-0.08854400	-0.31248400	1.88002100
H	-0.31348500	2.08712100	1.52067800
H	1.27214600	2.99039700	-0.25133100
H	1.07762200	1.48070100	-1.20127600

4d-TS-S$_N$2:
E = -1099.84
H = -1052.14
G = -1080.20
***N*imag** = 1, -161 cm^{-1}

C	0.62843000	-0.15724100	0.00466500
C	0.79459500	1.19415600	0.48406100
C	0.92831400	2.28772400	-0.29156500
I	3.34891200	-1.07159200	0.00084400
I	-2.23234900	-0.38493300	0.00123900
H	0.60163300	-0.37470700	-1.05146900
H	0.52763400	-0.97908100	0.68924200
H	0.80999300	1.31516800	1.56588700
H	1.04974100	3.27524700	0.14419500
H	0.91969300	2.22248100	-1.37630900

4d-TS-a-S$_N$2′:
E = -1100.36
H = -1052.57
G = -1080.47
***N*imag** = 1, -100 cm^{-1}

C	0.68333500	-0.15219300	0.06797400
C	0.78652000	1.14622500	0.55589800
C	0.86667700	2.28112200	-0.24426600
I	3.13861500	-1.51975100	-0.18245700
I	-1.60175200	3.55339100	-0.74197200
H	0.50778500	-0.32403800	-0.98794100
H	0.43150000	-0.96981000	0.73005500
H	0.80594300	1.28337300	1.63629300
H	1.13822500	3.23700400	0.18331700
H	1.00419600	2.18294600	-1.31512700

4d-TS-s-S_N2′:
E = -1097.39
H = -1049.69
G = -1077.35
***N*imag** = 1, -94 cm^{-1}

C	-0.20313400	0.27379200	-0.07809000
C	0.48289200	1.42501300	0.31614100
C	1.70734100	1.83266500	-0.21865900
I	-2.39080090	0.53301940	-1.64433509
I	1.74680408	3.90816135	-1.94983391
H	0.25712800	-0.39659800	-0.79584600
H	-0.89047600	-0.18917200	0.61779100
H	0.04867900	2.02890800	1.11206700
H	2.35582300	2.45988300	0.37893500
H	2.20835200	1.19523300	-0.93915400

1b-RC-S_N2:
E = -1164.06
H = -1117.11
G = -1141.70
***N*imag** = 0

C	0.03656500	0.32210200	-0.05970100
C	1.45860400	0.67357100	0.05731200
C	2.15520500	1.47043200	-0.77584700
Cl	-0.19917600	-1.49369900	0.15631000
F	-1.45794900	1.60433800	1.64554600
H	-0.36269400	0.53704200	-1.05539900
H	-0.65033300	0.87890100	0.77423000
H	1.95148900	0.31931300	0.96481800
H	3.16443500	1.79936100	-0.53637900
H	1.71780300	1.86006000	-1.69332000

1b-RC-S_N2′:
E = -1163.35
H = -1115.04
G = -1139.98
***N*imag** = 0

C	-0.34005900	1.05227200	-0.32740700
C	0.57133500	0.84010400	0.83383300
C	1.78331000	1.40931200	0.87753700
Cl	-0.99047300	-0.57131800	-0.93765700
F	1.23831200	2.69206100	-1.94129700
H	0.18993000	1.59745100	-1.16022100
H	-1.25163700	1.58277500	-0.03188000
H	0.20927700	0.22846200	1.66446100
H	2.42793000	1.28715600	1.74832300
H	2.11224300	2.01442400	0.02989100

1b-TS-S_N2:
E = -1158.20
H = -1110.18
G = -1134.52
***N*imag** = 1, -220.61169 cm^{-1}

C	0.77301400	-0.23329500	-0.01471600
C	0.76076600	1.15373700	0.46545300
C	0.94295300	2.24603200	-0.29174200
Cl	2.82020700	-0.79041600	-0.11040500
F	-1.42964500	-0.10359100	0.19826800
H	0.54255800	-0.41179500	-1.05383600
H	0.48118900	-1.00950100	0.66866600
H	0.59576600	1.27127300	1.53454800
H	0.90168600	3.24392800	0.13796700
H	1.11453700	2.17774700	-1.36342700

1b-TS-a-S_N2′:
E = -1156.52
H = -1108.48
G = -1133.07
***N*imag** = 1, -128.41593 cm^{-1}

C	-0.62809400	0.84784800	-0.52945700
C	0.45192900	1.08172100	0.43444400
C	1.72390200	1.32817100	0.05594900
Cl	-1.31009000	-0.94072200	-0.52206400
F	2.54437500	3.41247600	-1.10368500
H	-0.31040500	0.99417700	-1.56072600
H	-1.54162400	1.40939700	-0.32673500
H	0.16538700	1.13528300	1.48546400
H	2.48873500	1.57163500	0.78521600
H	2.05573800	1.35158700	-0.97499700

1b-TS-s-S_N2′:
E = -1154.87
H = -1106.90
G = -1131.11
***N*imag** = 1, -64.10978 cm^{-1}

C	-0.30780100	0.21975800	-0.12582100
C	0.42124400	1.40148800	0.33534900
C	1.62166100	1.78478200	-0.16138100
Cl	-1.94440900	0.57828400	-1.07187500
F	2.22556200	3.56161700	-1.70418700
H	0.25695300	-0.37645600	-0.84279200
H	-0.69521900	-0.40933800	0.67807500
H	-0.04584600	1.98143400	1.13187900
H	2.18159500	2.62324100	0.22793000
H	2.11201000	1.23663300	-0.95779800

1b-PC-S_N2:
E = -1184.03
H = -1134.37
G = -1160.57
***N*imag** = 0

C	0.08244500	0.17934000	-0.17847400
C	0.42529600	1.51117200	0.39899800
C	0.59800400	2.63426700	-0.30434000
Cl	3.09832900	-1.87623000	0.02472300
F	-1.10767600	-0.30917400	0.43736700
H	-0.11910500	0.24184200	-1.25425600
H	0.88719800	-0.55272500	0.00106600
H	0.58608200	1.52014300	1.47745000
H	0.91472500	3.56128200	0.16819100
H	0.46672000	2.65562800	-1.38437500

1b-PC-S_N2':
E = -1185.29
H = -1135.52
G = -1161.53
***N*imag** = 0

C	-0.64138000	1.79968900	0.12016100
C	0.60249300	2.28636400	0.17860900
C	1.78141400	1.59767800	-0.42883600
Cl	-0.36627700	-1.57734600	-1.59703100
F	2.55311500	2.52816700	-1.18896600
H	-0.85987400	0.86433100	-0.39697600
H	-1.46139600	2.31624500	0.61742700
H	0.81820500	3.21318500	0.71508800
H	2.46284000	1.21530400	0.34479400
H	1.48018500	0.77562000	-1.08439700

1c-RC-S_N2:
E = -1151.38
H = -1104.76
G = -1130.19
***N*imag** = 0

C	0.44415600	-0.03334800	-0.16293100
Br	-1.30610900	-0.81529300	0.47096800
C	0.60385400	1.32470600	0.36482500
C	0.91464800	2.42413300	-0.35043400
F	2.38892800	-1.61218700	0.49680300
H	0.34612500	-0.05063300	-1.25191900
H	1.32354500	-0.80285300	0.19917600
H	0.56154900	1.41348700	1.45210400
H	1.16760500	3.36504500	0.13437600
H	0.97664700	2.39998700	-1.43687000

1c-RC-S_N2':
E = -1150.58

H = -1102.58
G = -1128.31
***N*imag** = 0

C	0.80742000	0.07814400	0.15187200
C	1.35912500	1.19476700	0.96053900
C	1.08183100	2.47193700	0.66016900
Br	2.31836300	-1.14613300	-0.44456100
F	-0.72632300	1.40581200	-1.71350300
H	0.24609600	0.45237800	-0.75729500
H	0.20888300	-0.61279900	0.75254500
H	1.97344200	0.95051100	1.83086900
H	1.45067800	3.28626900	1.28389600
H	0.45815900	2.68753100	-0.20871600

1c-TS-S$_N$2:
E = -1147.01
H = -1099.21
G = -1124.45
***N*imag** = 1, -87.47533 cm^{-1}

C	0.44767700	-0.18997500	-0.11559500
Br	-1.67495800	-0.37094800	-0.19670100
C	0.80154000	1.13823600	0.41427300
C	1.00618800	2.23844100	-0.32589100
F	2.75310300	-0.85627700	0.72428100
H	0.70284600	-0.34874000	-1.15908200
H	0.75567200	-1.01371900	0.51588300
H	0.90618300	1.19216900	1.49518700
H	1.29028200	3.18372600	0.13037700
H	0.92896300	2.22122100	-1.41114500

1c-TS-a-S$_N$2′:
E = -1144.65
H = -1096.87
G = -1122.34
***N*imag** = 1, -161.86614 cm^{-1}

C	0.83423000	-0.20065900	0.03846000
C	1.06163000	1.10232800	0.63960200
C	0.99876200	2.25454600	-0.06719800
Br	2.59461100	-1.38891600	-0.16132000
F	-1.06966800	3.12242500	-1.26204800
H	0.48550800	-0.16062600	-0.99062100
H	0.25690300	-0.90198500	0.63812000
H	1.22200200	1.12440400	1.71834100
H	1.10006800	3.21312900	0.43280300
H	0.76781600	2.31427400	-1.12610100

1c-TS-s-S$_N$2′:
E = -1143.32
H = -1095.58

G = -1120.78
***N*imag** = 1, -119.76125 cm^{-1}

C	-0.27072900	0.22898400	-0.07786200
C	0.45538100	1.42100000	0.30878300
C	1.65268100	1.78182800	-0.21988600
Br	-2.08666300	0.56091600	-1.19119900
F	2.27023800	3.73482700	-1.57203400
H	0.25965100	-0.42300700	-0.76931200
H	-0.72935400	-0.32702100	0.73801200
H	0.01154200	2.03365800	1.09386600
H	2.22396300	2.64102700	0.10730300
H	2.11166700	1.19850100	-1.01248500

1c-PC-S_N2:
E = -1176.25
H = -1126.51
G = -1153.78
***N*imag** = 0

C	1.04264900	-0.15150700	-0.12232100
Br	-2.66233000	-1.30004600	0.44533800
C	1.13458100	1.22628600	0.44318100
C	1.18054000	2.34930600	-0.27871700
F	2.13077500	-0.93509400	0.35925800
H	1.10988600	-0.14865500	-1.21610800
H	0.10844200	-0.64556700	0.18211900
H	1.11523000	1.28366800	1.53172300
H	1.18242000	3.33012500	0.19094100
H	1.17817600	2.32757100	-1.36657200

1c-PC-S_N2′:
E = -1177.36
H = -1127.55
G = -1154.58
***N*imag** = 0

C	-0.11423600	0.33591600	0.88935000
C	-0.25790900	1.62361800	0.56111500
C	0.50569200	2.28491500	-0.53998500
Br	2.90695900	-1.18580800	-1.32804000
F	-0.38137400	3.03034400	-1.37127700
H	0.57248800	-0.31718500	0.35143800
H	-0.66173900	-0.08616500	1.73058200
H	-0.93396200	2.27154300	1.12304600
H	1.22396500	3.01692000	-0.14399100
H	1.03663000	1.56034900	-1.16329000

1d-RC-S_N2:
E = -1140.42
H = -1094.41
G = -1120.40

***N*imag** = 0

C	-0.00536400	0.19087900	-0.30136500
I	-2.06966900	-0.56249700	0.02767100
C	0.12597400	1.55309100	0.21398800
C	0.60660300	2.61821800	-0.46197700
F	1.65319200	-1.45839400	0.74584600
H	0.09281400	0.14495900	-1.39027600
H	0.75660500	-0.63838900	0.25175400
H	-0.08593300	1.67891200	1.27782800
H	0.82791800	3.55747400	0.04119000
H	0.83981900	2.56303500	-1.52375500

1d-RC-S_N2′:

E = -1139.24
H = -1091.58
G = -1117.85
***N*imag** = 0

C	0.80747500	0.06604500	0.10906500
C	1.31621200	1.19975000	0.91553400
C	1.04058700	2.47176800	0.58732900
I	2.52452700	-1.21276200	-0.57541800
F	-0.75308700	1.21426500	-1.78919000
H	0.21742600	0.40461300	-0.80894500
H	0.27127900	-0.66868100	0.71637400
H	1.89677600	0.98303000	1.81586100
H	1.37786300	3.29722800	1.21356000
H	0.44887200	2.67704300	-0.30393800

1d-TS-S_N2:

E = -1136.25
H = -1088.68
G = -1114.54
***N*imag** = 1, -105.64665 cm^{-1}

C	0.27980700	-0.20207300	-0.05967100
I	-2.05585300	-0.56335400	0.04435200
C	0.57265600	1.15844800	0.40550000
C	0.72057100	2.23636300	-0.38111500
F	2.61279500	-0.89212100	0.65584800
H	0.44113500	-0.37619700	-1.11947100
H	0.68053500	-0.99283800	0.56606900
H	0.69859300	1.26404200	1.48107200
H	0.97461600	3.20928600	0.03257600
H	0.61727300	2.17407900	-1.46242900

1d-TS-a-S_N2′:

E = -1133.73
H = -1086.18
G = -1112.29
***N*imag** =1, -174.49526 cm^{-1}

C	0.82112700	-0.16756500	0.04828600
C	1.04923800	1.12667200	0.64535700
C	0.94744100	2.28701500	-0.04906300
I	2.80506900	-1.45432500	-0.32807400
F	-1.14409800	3.01135000	-1.32171600
H	0.42297000	-0.14651400	-0.96254600
H	0.34331400	-0.91428700	0.67701000
H	1.25695200	1.14448100	1.71626100
H	1.04823100	3.23863900	0.46602500
H	0.66687000	2.35773600	-1.09780800

1d-TS-s-S$_N$2′:
E = -1132.74
H = -1085.22
G = -1111.14
***N*imag** = 1, -145.02934 cm^{-1}

C	-0.11132400	0.18512400	0.05289300
C	0.57781200	1.41422400	0.33863100
C	1.72277500	1.80671800	-0.28269500
I	-2.23035700	0.38184400	-1.12467700
F	2.13938600	3.88229400	-1.54090900
H	0.38220000	-0.48808400	-0.64379000
H	-0.55540300	-0.33122900	0.89982200
H	0.16719000	2.03798300	1.13281800
H	2.27585600	2.70468800	-0.02981300
H	2.13731900	1.21783500	-1.09696200

1d-PC-S$_N$2:
E = -1169.51
H = -1119.69
G = -1147.95
***N*imag** = 0

C	1.16352700	-0.14871300	0.12010200
I	-2.97000200	-1.45460800	0.14557300
C	1.11479000	1.29937400	0.47953900
C	1.16981500	2.30436200	-0.39782800
F	2.23414700	-0.77696900	0.81553000
H	1.34158100	-0.29852800	-0.95020400
H	0.23614400	-0.66190300	0.40406200
H	0.98320800	1.51252000	1.54048800
H	1.06983000	3.34064200	-0.08445700
H	1.27723700	2.12370500	-1.46519500

1d-PC-S_N2':
E = -1170.44
H = -1120.58
G = -1148.46
***N*imag** = 0

C	-0.17695400	0.36623000	0.88977900
C	-0.30661800	1.65600000	0.56563000
C	0.42891600	2.30896400	-0.55911400
I	3.12464900	-1.48283800	-1.40124700
F	-0.47428300	3.05695200	-1.36608100
H	0.47651000	-0.30374600	0.33424000
H	-0.70209400	-0.04693600	1.74875200
H	-0.95122900	2.31405100	1.15138100
H	1.16576200	3.03427800	-0.18627900
H	0.93473800	1.58059800	-1.19836600

2c-RC-S_N2:
E = -1132.53
H = -1083.85
G = -1111.37
***N*imag** = 0

C	0.22877900	0.03076600	-0.26727800
Br	-1.60811700	-0.60759600	0.25000100
C	0.51004100	1.33733500	0.36919400
C	0.65094800	2.49456400	-0.28875000
Cl	3.02778700	-1.92786700	0.49688700
H	0.19432700	0.08258200	-1.35523300
H	0.91917900	-0.76006600	0.06699100
H	0.65788600	1.31169000	1.44793100
H	0.92031000	3.41219000	0.22861700
H	0.52860200	2.55688700	-1.36811000

2c-RC-S_N2':
E = -1133.12
H = -1084.29
G = -1111.62
***N*imag** = 0

C	0.09450700	0.10500700	-0.31075000
C	-0.14824900	1.37127100	0.42401800
C	0.51015500	2.50381700	0.14692200
Br	-1.61569700	-0.59065400	-1.11791500
Cl	3.06498400	1.09803400	-2.31341200
H	0.80477300	0.21306800	-1.13545600
H	0.38543100	-0.71743500	0.34560800
H	-0.87290100	1.33720600	1.23887500
H	0.33884100	3.40123400	0.73934500
H	1.25365200	2.53992500	-0.64796600

2c-TS-S$_N$2:
E = -1126.25
H = -1078.35
G = -1104.41
***N*imag** = 1, -229 cm^{-1}

C	0.59849500	-0.20632000	-0.00897100
Br	-1.96355500	-0.32804800	-0.00741800
C	0.77914200	1.14870000	0.47674600
C	0.92736500	2.23731700	-0.29718500
Cl	3.01623600	-0.90165700	-0.01346200
H	0.59628000	-0.41823400	-1.06513700
H	0.53340800	-1.03215100	0.67286400
H	0.79584000	1.26425300	1.55903100
H	1.06571200	3.22357500	0.13741300
H	0.91951700	2.17079000	-1.38192000

2c-TS-a-S$_N$2′:
E = -1126.68
H = -1078.66
G = -1104.49
***N*imag** = 1, -145 cm^{-1}

C	0.49950182	0.23012007	0.82421312
C	0.19189886	1.24739056	-0.06581328
C	1.13478231	1.86904757	-0.89422156
Cl	1.04789969	0.98137381	3.10584083
Br	1.27349167	0.93795832	-3.17733909
H	1.47130916	-0.24789352	0.78714905
H	-0.28539295	-0.33312988	1.31083615
H	-0.83885034	1.59556674	-0.11589530
H	0.90414137	2.82577649	-1.34576995
H	2.18873540	1.68258884	-0.72031898

2c-TS-s-S$_N$2′:
E = -1123.74
H = -1175.78
G = -1101.36
***N*imag** = 1, -139.76634 cm^{-1}

C	-0.16247100	0.26310300	-0.05024800
C	0.53279100	1.41478100	0.35742900
C	1.75190000	1.81986800	-0.17536100
Br	-2.12242100	0.53774800	-1.43418900
Cl	1.79834000	3.64915100	-1.73333800
H	0.31773300	-0.40340500	-0.75958400
H	-0.80615800	-0.22667800	0.67033000
H	0.09814600	2.01323600	1.15715900
H	2.39508700	2.46520400	0.40783200
H	2.25169000	1.19148300	-0.90414600

2c-PC-S_N2:
E = -1137.65
H = -1088.64
G = -1116.35
***N*imag** = 0

C	1.20314600	-0.03496200	-0.27810700
Br	-2.30729900	-1.28064000	0.34225000
C	1.24361600	1.29999900	0.37575600
C	1.35479700	2.46297800	-0.27396800
Cl	2.65528500	-1.03820500	0.21946400
H	1.25248300	0.03253500	-1.36564400
H	0.31269000	-0.60751200	0.01809000
H	1.12568700	1.29554700	1.45851400
H	1.31860100	3.41315000	0.25322700
H	1.45552600	2.50561700	-1.35643900

2c-PC-S_N2':
E = -1138.3
H = -1089.17
G = -1116.72
***N*imag** = 0

C	0.76968900	0.05159100	-0.23388600
C	2.01974200	0.71594400	0.23359200
C	2.06889800	2.00154600	0.59837800
Cl	1.02372600	-0.76625600	-1.85738700
Br	-1.86234500	2.99827500	0.56994200
H	-0.06010800	0.75250000	-0.34910100
H	0.46913500	-0.76595600	0.42702100
H	2.90797600	0.08601400	0.29921200
H	2.99337700	2.43360400	0.97754700
H	1.18555700	2.63638700	0.55811700

2d-RC-S_N2:
E = -1120.7
H = -1072.27
G = -1100.36
***N*imag** = 0

C	0.24383900	0.05260700	-0.28747000
I	-1.80604600	-0.64915200	0.22658600
C	0.52327200	1.34920300	0.36104400
C	0.63736900	2.52125500	-0.27844000
Cl	2.99215100	-1.88924600	0.53038200
H	0.22175500	0.10518800	-1.37543200
H	0.90537100	-0.75586800	0.06231100
H	0.69652100	1.30852300	1.43550400
H	0.90666200	3.43156800	0.25146000
H	0.48774200	2.60376000	-1.35298800

2d-RC-S_N2':
E = -1121.03
H = -1072.45
G = -1100.31
***N*imag** = 0

C	0.50196600	-0.07725600	0.82140700
C	0.21299100	1.22967700	0.75208800
C	0.64233500	2.08661700	-0.37569000
Cl	2.95342200	-0.10254500	-2.14124900
I	-1.11357500	3.01687600	-1.38514500
H	1.06653700	-0.56857600	0.03149000
H	0.20854000	-0.66235700	1.69130700
H	-0.32959200	1.71432100	1.56497500
H	1.18537800	2.97747900	-0.05681800
H	1.18762900	1.54942100	-1.15917900

2d-TS-S_N2:
E = -1117.03
H = -1069.22
G = -1095.92
***N*imag** = 1, -190.96801 cm^{-1}

C	0.56552800	-0.19251200	-0.00849500
I	-2.14428500	-0.39119100	-0.00485300
C	0.76730400	1.15738500	0.47620400
C	0.93593300	2.24448000	-0.29775500
Cl	3.06941600	-0.93863100	-0.01400600
H	0.59965800	-0.40904700	-1.06401900
H	0.55719000	-1.02363700	0.67130400
H	0.78997900	1.27345700	1.55839800
H	1.09893500	3.22642100	0.13770600
H	0.92490200	2.17942900	-1.38256200

2d-TS-a-S_N2':
E = -1117.26
H = -1069.36
G = -1095.9
***N*imag** = 1, -110.88866 cm^{-1}

C	0.73087500	-0.15090700	0.09795000
C	0.69932100	1.14470300	0.55799900
C	0.67493000	2.27825700	-0.28179000
Cl	3.12744000	-1.16551200	-0.26541500
I	-1.68896600	3.30114800	-0.66553500
H	0.59541200	-0.37342800	-0.95366900
H	0.67022300	-0.99027800	0.77665900
H	0.72729100	1.31535200	1.63340300
H	1.00439500	3.23194400	0.11277500
H	0.85891400	2.13662800	-1.34154700

2d-TS-s-S$_N$2′:
E = -1114.35
H = -1066.53
G = -1092.81
***N*imag** = 1, -113.62752 cm^{-1}

C	-0.15440900	0.25132400	-0.04639100
C	0.54224300	1.40958500	0.36265500
C	1.75779800	1.80948900	-0.15680300
I	-2.27355100	0.55738500	-1.49749200
Cl	1.86707100	3.73291400	-1.80707600
H	0.32974200	-0.41237600	-0.75609000
H	-0.78132800	-0.24744300	0.68316200
H	0.10200900	2.00567000	1.16109300
H	2.37372100	2.51424300	0.38364300
H	2.25888900	1.21202200	-0.90926600

2d-PC-S$_N$2:
E = -1130.59
H = -1081.47
G = -1110.09
***N*imag** = 0

C	1.41339000	0.03230800	-0.28966200
I	-2.52776500	-1.17768900	0.29132000
C	1.46701400	1.36010600	0.37939900
C	1.55946300	2.52841700	-0.26241900
Cl	2.87802600	-0.97496900	0.15434300
H	1.42782400	0.11238900	-1.37705200
H	0.53900400	-0.54812800	0.02363100
H	1.38190500	1.34432600	1.46496300
H	1.53970500	3.47308400	0.27495800
H	1.62738400	2.58148700	-1.34682300

2d-PC-S$_N$2′:
E = -1131.14
H = -1081.94
G = -1110.36
***N*imag** = 0

C	1.23345000	-0.12739600	-0.11885200
C	1.85632300	0.89597600	0.76804800
C	1.59089600	2.20324100	0.68028500
Cl	2.50817900	-1.15237900	-0.94531500
I	-1.91732200	2.41184900	-1.99466800
H	0.61977700	0.31451900	-0.90489200
H	0.63714900	-0.84933800	0.44535100
H	2.53242000	0.51971700	1.53651600
H	2.03534300	2.90689600	1.38122300
H	0.90337800	2.59911900	-0.06369700

3d-RC-S$_N$2:
E = -1112.59
H = -1064.07
G = -1093.22
***N*imag** = 0

C	0.20094885	0.41164493	-0.19478817
Br	-1.32823660	3.60505436	-1.26763581
C	1.45430611	0.87950625	0.43003179
C	2.68619852	0.55300657	0.01708929
I	-0.83380888	-1.05907226	1.12461672
H	0.35250528	-0.14669749	-1.11744609
H	-0.53726221	1.21086852	-0.33002732
H	1.33518063	1.57762104	1.25711692
H	3.57061217	0.97621850	0.48643736
H	2.84933413	-0.12763640	-0.81582969

3d-RC-S$_N$2′:
E = -1117.73
H = -1069.38
G = -1096.61
***N*imag** = 0

C	-0.32050400	0.19786700	0.94404800
I	3.11600300	-0.55183200	-1.71974300
C	-0.52625100	1.51111100	0.78941100
C	0.08113700	2.30600600	-0.30623900
Br	-1.33466800	3.20859600	-1.40918000
H	0.30613100	-0.36680400	0.25782700
H	-0.74864600	-0.33386500	1.79125200
H	-1.13761400	2.05937000	1.50668000
H	0.66810800	3.15104900	0.05739500
H	0.66832600	1.70850700	-1.00374700

3d-TS-S$_N$2:
E = -1108.47
H = -1060.72
G = -1088.21
***N*imag** = 1, -173.29195 cm^{-1}

C	0.66674300	-0.19753100	-0.00800500
Br	-2.05099300	-0.36389500	-0.01205600
C	0.80830800	1.15807300	0.47563100
C	0.92010900	2.25409400	-0.29777700
I	3.30220900	-1.06532000	-0.00989200
H	0.60337300	-0.40518100	-1.06414600
H	0.51003800	-1.01253600	0.67355200
H	0.81726200	1.27641800	1.55779100
H	1.01450400	3.24427900	0.13898400
H	0.91504500	2.18959600	-1.38260800

3d-TS-a-S$_N$2′:
E = -1108.89
H = -1061.04
G = -1088.36
***N*imag** =1, -104.55722 cm^{-1}

C	0.55379900	-0.04979300	0.07504500
I	2.98625400	-1.15384900	-0.14324100
C	0.54783500	1.26168600	0.57370800
C	0.50072500	2.39165500	-0.21873500
Br	-1.99014000	3.33788000	-0.72914300
H	0.34819700	-0.21343800	-0.97712900
H	0.29869900	-0.87305700	0.73025500
H	0.56226100	1.39104700	1.65513000
H	0.62003900	3.37802900	0.20805200
H	0.61253400	2.31803900	-1.29411700

3d-TS-s-S$_N$2′:
E = -1105.94
H = -1058.17
G = -1085.22
***N*imag** =1, -101.18496 cm^{-1}

C	-0.18859600	0.27271700	-0.05983400
C	0.49048300	1.42068400	0.31838200
C	1.72064600	1.82956700	-0.22265800
Br	-2.32193200	0.46514700	-1.58974800
I	1.74129800	3.89394600	-1.89511100
H	0.23996200	-0.39848800	-0.79518800
H	-0.93218800	-0.15352500	0.59911800
H	0.06101600	2.03074800	1.11215100
H	2.37885700	2.43306500	0.38946100
H	2.21839200	1.18311000	-0.93802600

3d-PC-S$_N$2:
E = -1117.32
H = -1068.47
G = -1097.93
***N*imag** = 0

C	-0.01504200	0.16432200	-0.30003700
Br	-1.86812800	-0.47581100	0.14844800
C	0.26421600	1.45348500	0.37379800
C	0.40652400	2.62387300	-0.25805100
I	3.50359500	-1.86057100	0.40310800
H	-0.01034000	0.23554300	-1.38672600
H	0.64457000	-0.64016500	0.03546700
H	0.39775700	1.40387400	1.45318400
H	0.66354400	3.53113400	0.28245200
H	0.29893400	2.70818700	-1.33724700

3d-PC-S_N2':
E = -1112.81
H = -1064.18
G = -1093.06
***N*imag** = 0

C	0.38908500	0.16478800	-0.28208000
I	0.80846300	-0.69777400	-2.29172200
C	1.53768400	0.95964000	0.20685400
C	1.45055400	2.26223700	0.50886400
Br	-2.63108500	2.51335100	0.41841300
H	-0.53443700	0.73837500	-0.39625900
H	0.21558700	-0.74263800	0.29770800
H	2.47883800	0.42799700	0.35220100
H	2.31014700	2.79565200	0.91013900
H	0.51538700	2.80613700	0.39794200

Table S5. Cartesian coordinates (in Å), energies (*E*, *H*, and *G*, in kcal mol^{-1}), and number of imaginary vibrational frequencies (N_{imag}) of all stationary points in solution, computed at COSMO(DCM)-ZORA-OLYP/QZ4P. Not all reactions are shown because many stationary points are shared over two reaction systems due to symmetry (*e.g.*, reaction 1b = 2a).

3-fluoroprop-1-ene:
E = −1093.33
H = −1044.28
G = −1064.82
N_{imag} = 0

C	0.382048	-0.773813	2.125443
C	-0.272392	-0.389715	1.025500
C	0.289520	0.546500	0.008387
H	-0.338117	1.436483	-0.113594
H	-0.078945	-1.428100	2.860284
H	1.394302	-0.435623	2.335296
H	-1.287609	-0.740181	0.841029
H	1.314496	0.846862	0.242490
F	0.322860	-0.084216	-1.273634

3-chloroprop-1-ene:
E = −1051.65
H = −1004.25
G = −1025.54
N_{imag} = 0

C	0.447812	-0.651030	2.190159
C	-0.312910	-0.367271	1.127922
C	0.170097	0.399069	-0.054438
Cl	0.116819	-0.615446	-1.577288
H	0.042961	-1.180565	3.048056
H	1.493354	-0.356133	2.241786
H	-1.356422	-0.677909	1.104842
H	1.204756	0.724341	0.050851
H	-0.467794	1.256529	-0.276659

3-bromoprop-1-ene:
E = −1038.00
H = −990.84
G = −1012.95
N_{imag} = 0

Br	0.282446	-0.481256	-1.742414
C	0.388463	-0.745602	2.142929
C	-0.321128	-0.339340	1.083319
C	0.229235	0.507957	-0.002616
H	-0.399426	1.369185	-0.228760
H	-0.067350	-1.334685	2.933721
H	1.440731	-0.496060	2.257871
H	-1.372638	-0.609426	0.998295
H	1.257714	0.819276	0.171652

3-iodoprop-1-ene:
E = −1025.28
H = −978.36
G = −1001.04
N_{imag} = 0

C	0.441526	-0.813033	2.090874
C	-0.311567	-0.343969	1.087184
C	0.182512	0.574072	0.035676
H	-0.479618	1.422006	-0.136674
H	0.019544	-1.456638	2.857544
H	1.496699	-0.565147	2.180638
H	-1.363631	-0.620040	1.030019
H	1.205570	0.909881	0.194852
I	0.227069	-0.392639	-1.946694

Fluoride:
E = −182.05
H = −181.16
G = −190.86
N_{imag} = 0

F	0.00000000	0.00000000	0.00000000

Chloride:
E = −154.02
H = −153.13
G = −163.40
N_{imag} = 0

Cl	0.00000000	0.00000000	0.00000000

Bromide:
E = −141.56
H = −140.67
G = −151.58
N_{imag} = 0

Br	0.00000000	0.00000000	0.00000000

Iodide:
E = −127.30
H = −126.41
G = −137.69
N_{imag} = 0

I	0.00000000	0.00000000	0.00000000

1a-TS-S_N2:
E = −1250.25
H = −1201.70
G = −1225.10
***N*imag** = 1, -449.428 cm^{-1}

C	0.620124	-0.222655	0.008422
F	-1.330092	-0.182857	0.001671

F	2.521378	-0.658331	-0.007372
C	0.790476	1.144700	0.498013
C	0.918955	2.217939	-0.294466
H	0.593133	-0.419627	-1.050568
H	0.520557	-1.043250	0.695123
H	0.810365	1.267774	1.579520
H	1.043579	3.214836	0.119021
H	0.903475	2.131870	-1.378343

1a-TS-a-S_N2':
E = -1249.92
H = -1201.24
G = -1224.35
***N*imag** =1, -381.121 cm^{-1}

C	0.829389	-0.158160	0.054274
C	0.781456	1.147210	0.569086
C	0.721983	2.285146	-0.251327
F	2.492208	-0.765342	-0.129813
F	-0.945263	2.827159	-0.556215
H	0.566394	-0.299032	-0.991353
H	0.536555	-0.982395	0.699131
H	0.789411	1.282765	1.649593
H	1.020901	3.242921	0.166135
H	0.970085	2.164107	-1.303060

1b-TS-S_N2:
E = -1216.96
H = -1168.68
G = -1193.13
***N*imag** = 1, -364.953 cm^{-1}

C	0.664850	-0.185069	0.007879
C	0.811593	1.172990	0.505248
C	0.888226	2.255322	-0.285660
Cl	2.885435	-0.888995	-0.007974
F	-1.474542	-0.162266	-0.030011
H	0.582649	-0.369761	-1.050870
H	0.463641	-0.996174	0.683276
H	0.844064	1.291000	1.586555
H	0.974476	3.254323	0.131694
H	0.862640	2.172749	-1.369359

1b-TS-a-S_N2':
E = -1215.58
H = -1167.22
G = -1191.58
***N*imag** =1, -303.412 cm^{-1}

C	-0.591996	1.031234	-0.539071
C	0.461395	1.309513	0.353341
C	1.778809	1.376363	-0.049999
Cl	-1.187743	-1.153810	-0.537653

F	2.308482	3.281782	-0.646669
H	-0.382872	1.036932	-1.603586
H	-1.602430	1.316257	-0.270766
H	0.215495	1.507229	1.394795
H	2.575177	1.458033	0.678685
H	2.065536	1.028042	-1.035668

1c-TS-S_N2:
E = -1209.60
H = -1161.47
G = -1186.60
***N*imag** = 1, -81.463 cm^{-1}

C	0.447677	-0.189975	-0.115595
Br	-1.674958	-0.370948	-0.196701
C	0.801540	1.138236	0.414273
C	1.006188	2.238441	-0.325891
F	2.753103	-0.856277	0.724281
H	0.702846	-0.348740	-1.159082
H	0.755672	-1.013719	0.515883
H	0.906183	1.192169	1.495187
H	1.290282	3.183726	0.130377
H	0.928963	2.221221	-1.411145

1c-TS-a-S_N2′:
E = -1204.13
H = -1155.90
G = -1181.14
***N*imag** =1, -264.471 cm^{-1}

C	0.696442	-0.148133	0.051351
C	0.862100	1.149106	0.572571
C	0.939886	2.269043	-0.227111
Br	2.785767	-1.356511	-0.160953
F	-0.953116	3.086958	-0.616865
H	0.463553	-0.260028	-1.002190
H	0.315070	-0.933764	0.692469
H	0.898297	1.267888	1.653723
H	1.181132	3.235358	0.196307
H	1.062732	2.169004	-1.299264

1d-TS-S_N2:
E = -1205.20
H = -1157.67
G = -1182.83
***N*imag** = 1, -37.941 cm^{-1}

C	0.209140	-0.065673	0.160291
I	-1.880481	-0.779908	0.093281
C	0.280448	1.377478	0.480263
C	0.706716	2.317360	-0.374165
F	3.185901	-1.905050	-0.334659
H	0.617968	-0.324255	-0.813203

H	0.649320	-0.700778	0.926700
H	-0.010850	1.663463	1.490284
H	0.778066	3.359467	-0.075166
H	1.005900	2.073532	-1.390895

1d-TS-a-S$_N$2′:
E = -1191.44
H = -1143.33
G = -1169.19
***N*imag** =1, -252.280 cm^{-1}

C	0.703950	-0.136530	0.035920
C	0.851966	1.157157	0.567300
C	0.884651	2.291193	-0.216544
I	2.995696	-1.430494	-0.265779
F	-1.047864	3.059288	-0.565136
H	0.458153	-0.248193	-1.014723
H	0.367509	-0.941906	0.677392
H	0.909930	1.263467	1.648926
H	1.102880	3.257653	0.218624
H	0.990243	2.211567	-1.292247

2b-TS-S$_N$2:
E = -1185.91
H = -1137.78
G = -1163.22
***N*imag** = 1, -301.202 cm^{-1}

C	0.628704	-0.155481	0.017096
C	0.794790	1.189488	0.511608
C	0.925090	2.264682	-0.288481
Cl	3.032567	-0.940135	-0.035983
Cl	-1.893784	-0.335537	-0.025604
H	0.602063	-0.353838	-1.042974
H	0.528149	-0.986148	0.692171
H	0.812659	1.315527	1.591903
H	1.048465	3.262055	0.122783
H	0.911621	2.173688	-1.371493

2b-TS-a-S$_N$2′:
E = -1184.68
H = -1136.51
G = -1161.98
***N*imag** =1, -229.226 cm^{-1}

C	0.755731	-0.142999	0.064102
C	0.784611	1.147928	0.574728
C	0.794804	2.272214	-0.240106
Cl	3.067462	-1.107407	-0.201989
Cl	-1.527082	3.147824	-0.672558
H	0.600752	-0.307314	-0.995995
H	0.582624	-0.990971	0.713499
H	0.799723	1.283616	1.653811

H	0.982921	3.253902	0.174201
H	0.919853	2.168470	-1.311742

2c-TS-S_N2:
E = -1174.46
H = -1126.41
G = -1152,67
***N*imag** = 1, -279.854 cm^{-1}

C	0.636716	-0.178682	0.005267
Br	-2.044506	-0.404006	-0.035410
C	0.792168	1.165949	0.501105
C	0.934362	2.243445	-0.295174
Cl	3.032883	-0.969608	-0.051737
H	0.603139	-0.375873	-1.055091
H	0.526034	-1.008997	0.679505
H	0.799637	1.291251	1.581695
H	1.055914	3.239246	0.120298
H	0.932093	2.155500	-1.378498

2c-TS-a-S_N2′:
E = -1173.37
H = -1125.38
G = -1151.54
***N*imag** =1, -213.911 cm^{-1}

C	0.499122	0.215698	0.808283
C	0.186334	1.273570	-0.036960
C	1.135433	1.908959	-0.825287
Cl	1.040279	0.998237	3.131511
Br	1.288502	0.871756	-3.285922
H	1.479900	-0.243838	0.766714
H	-0.283362	-0.361229	1.283264
H	-0.844275	1.619096	-0.079936
H	0.897324	2.826770	-1.346672
H	2.188260	1.679779	-0.706314

2d-TS-S_N2:
E = -1161.73
H = -1113.75
G = -1140.58
***N*imag** = 1, -271.902 cm^{-1}

C	0.657506	-0.175408	0.005766
I	-2.258856	-0.470221	-0.026114
C	0.800874	1.170410	0.498276
C	0.952454	2.248568	-0.297054
Cl	3.039297	-0.973211	-0.055055
H	0.611491	-0.374859	-1.054098
H	0.531427	-1.002473	0.681876
H	0.801385	1.297973	1.578747
H	1.072053	3.243888	0.120041
H	0.956929	2.161488	-1.380463

2d-TS-a-S$_N$2′:
E = -1160.74
H = -1112.73
G = -1139.55
***N*imag** =1, -208.137 cm^{-1}

C	0.768125	-0.169535	0.078034
C	0.793368	1.132730	0.567818
C	0.786324	2.248219	-0.255404
Cl	3.067936	-1.094258	-0.222876
I	-1.878678	3.310705	-0.731772
H	0.582507	-0.351701	-0.974278
H	0.609083	-1.005516	0.746275
H	0.825950	1.280703	1.645209
H	0.963989	3.236619	0.147943
H	0.881231	2.139940	-1.330119

3c-TS-S$_N$2:
E = -1163.09
H = -1115.11
G = -1142.18
***N*imag** = 1, -256.089 cm^{-1}

C	0.629693	-0.145033	0.017040
C	0.794805	1.199099	0.508479
C	0.927681	2.278174	-0.288428
Br	3.167734	-1.018219	-0.031754
Br	-2.044010	-0.378797	-0.029820
H	0.604559	-0.347478	-1.042797
H	0.528087	-0.974463	0.694028
H	0.810201	1.325896	1.588925
H	1.050121	3.273780	0.127137
H	0.917401	2.191198	-1.371773

3c-TS-a-S$_N$2′:
E = -1162.11
H = -1114.10
G = -1141.12
***N*imag** =1, -192.442 cm^{-1}

C	0.647701	-0.138824	0.071787
C	0.786436	1.148393	0.574756
C	0.902053	2.267993	-0.239007
Br	2.989661	-1.386849	-0.189888
Br	-1.451584	3.425753	-0.720682
H	0.485215	-0.297393	-0.988240
H	0.400213	-0.964084	0.726442
H	0.805898	1.285657	1.653885
H	1.169892	3.229453	0.178956
H	1.025910	2.155162	-1.310058

3d-TS-S_N2:
E = -1150.44
H = -1102.54
G = -1130.17
***N*imag** = 1, -241.962 cm^{-1}

C	0.614297	-0.163924	0.006144
Br	-2.044057	-0.414001	-0.048078
C	0.788939	1.178198	0.496496
C	0.910909	2.261845	-0.297497
I	3.364385	-1.157725	-0.033328
H	0.602795	-0.370189	-1.053548
H	0.530239	-0.995195	0.683759
H	0.811426	1.303617	1.577099
H	1.033242	3.255960	0.121575
H	0.894424	2.179411	-1.381149

3d-TS-a-S_N2′:
E = -1149.54
H = -1101.61
G = -1129.19
***N*imag** =1, -183.432 cm^{-1}

C	0.464658	-0.024289	0.088181
I	3.107709	-1.187003	-0.184960
C	0.485407	1.270653	0.585948
C	0.495911	2.398528	-0.227616
Br	-1.945864	3.312483	-0.715992
H	0.326929	-0.203764	-0.972293
H	0.300172	-0.867758	0.745941
H	0.490325	1.411435	1.664952
H	0.675010	3.379213	0.193137
H	0.639946	2.298702	-1.297472

4d-TS-S_N2:
E = -1137.90
H = -1090.08
G = -1118.27
***N*imag** = 1, -232.065 cm^{-1}

C	0.631356	-0.133037	0.017392
C	0.796342	1.211094	0.503155
C	0.929507	2.294895	-0.290112
I	3.359173	-1.137121	-0.028963
I	-2.258282	-0.446792	-0.027405
H	0.605399	-0.342689	-1.041669
H	0.529895	-0.960178	0.698043
H	0.811980	1.339350	1.583714
H	1.051468	3.288345	0.130572
H	0.919757	2.213353	-1.373937

4d-TS-a-S$_N$2′:
E = -1137.04
H = -1089.19
G = -1117.31
***N*imag** =1, -173.811 cm^{-1}

C	0.652840	-0.142226	0.074276
C	0.787765	1.148741	0.569516
C	0.897506	2.272201	-0.240227
I	3.175065	-1.513722	-0.209008
I	-1.641008	3.540474	-0.762742
H	0.488776	-0.309887	-0.984443
H	0.413814	-0.964844	0.735548
H	0.808492	1.287268	1.648831
H	1.157582	3.233848	0.182483
H	1.020212	2.166416	-1.312459

Table S6. Cartesian coordinates (in Å), energies (*E*, *H*, and *G*, in kcal mol^{-1}), and number of imaginary vibrational frequencies (N_{imag}) of all stationary points for MeZ^{-} + $H_2C{=}CHCH_2Y$ systems, computed at ZORA-OLYP/QZ4P.

MeO⁻:
E = −582.82
H = -559.30
G = -575.02
N_{imag} = 0

O	0.00000000	0.00000000	4.20632800
C	0.00000000	0.00000000	2.88158800
H	-0.51499500	0.89199700	2.37190900
H	1.02999000	0.00000000	2.37190900
H	-0.51499500	-0.89199700	2.37190900

MeS⁻:
E = −541.90
H = -517.51
G = -534.21
N_{imag} = 0

S	0.00000000	0.00000000	4.63831600
C	0.00000000	0.00000000	2.80917500
H	-0.51035600	0.88396300	2.39079300
H	1.02071200	0.00000000	2.39079300
H	-0.51035600	-0.88396300	2.39079300

MeSe⁻:
E = −528.56
H = -504.21
G = -521.83
N_{imag} = 0

Se	0.00000000	0.00000000	-0.28611500
H	0.51266700	-0.88796600	-2.66943100
H	-1.02533500	0.00000000	-2.66943100
C	0.00000000	0.00000000	-2.27856100
H	0.51266700	0.88796600	-2.66943100

MeTe⁻:
E = −517.53
H = -493.24
G = -511.47
N_{imag} = 0

Te	0.00000000	0.00000000	-0.08733200
H	0.51427300	-0.89074700	-2.66510400
H	-1.02854600	0.00000000	-2.66510400
C	0.00000000	0.00000000	-2.29365300
H	0.51427300	0.89074700	-2.66510400

MeO⁻-TS-S_N2:
E = −1672.30

H = -1599.54
G = -1626.65
***N*imag** = 1, -418.76753 cm^{-1}

O	0.05762400	0.28018200	-0.68023600
H	1.09818900	0.80876800	3.21954900
H	-1.23494900	0.76061900	-2.26567600
C	-0.19097500	0.43114200	-2.01448400
H	0.47600400	1.16835300	-2.53715900
H	-0.04679000	-0.52719300	-2.56558900
C	-0.22756600	2.07376700	0.25869000
H	-0.66216300	0.71172400	2.64434400
F	-0.57211900	3.78793600	0.90010400
C	0.68712900	1.70562000	1.34099300
C	0.35654900	1.04404900	2.45910400
H	-1.27296800	1.81795900	0.31834000
H	0.15082500	2.53492200	-0.63676600
H	1.72312500	2.01307300	1.20051700

MeO⁻-TS-a-S_N2′:
E = -1679.67
H = -1606.77
G = -1633.08
***N*imag** =1, -152.19219 cm^{-1}

O	0.32753600	0.52175500	-0.52203300
H	-0.88202100	2.29510300	0.17469900
H	-1.19078500	1.01378200	-1.88495900
C	-0.12808900	0.65339900	-1.80077700
H	0.46460400	1.36303200	-2.44176600
H	-0.10943000	-0.31657800	-2.34903200
C	1.92750800	2.46676000	2.18224400
C	0.56720600	2.22461800	1.74577200
C	0.17849300	2.24626600	0.41627500
F	2.16791200	3.86288200	2.75090700
H	0.82595100	2.73644200	-0.31001600
H	2.65644300	2.41425800	1.36726600
H	2.25856700	1.84920200	3.02680000
H	-0.15367800	1.90979100	2.50074600

MeS⁻-TS-S_N2:
E = -1625.12
H = -1552.86
G = -1581.36
***N*imag** = 1, -376.61471 cm^{-1}

S	0.32733700	-0.24412300	-0.82980700
H	1.14495000	1.01556900	3.34108900
H	-1.26120000	0.49490400	-2.54628400
C	-0.20132900	0.20823300	-2.51002900
H	0.38738100	1.04220600	-2.91660200
H	-0.06575300	-0.64806900	-3.18265200
C	-0.18298200	1.87628500	0.24192000

H	-0.60174700	0.77401300	2.76659500
F	-0.69100600	3.69025300	0.75573000
C	0.72542600	1.66408800	1.36392600
C	0.40554400	1.12231300	2.55112400
H	-1.21844600	1.58890700	0.31364800
H	0.16977200	2.33794500	-0.66178800
H	1.74981300	1.99679200	1.19993400

MeS⁻-TS-a-S_N2':

E = -1630.18

H = -1557.87

G = -1585.63

***N*imag** =1, -209.92350 cm^{-1}

S	0.55129600	0.05326700	-0.61945400
H	-0.79124500	2.00320000	0.17380400
H	-1.24751600	0.84589900	-2.06728600
C	-0.23107900	0.45095500	-2.20826500
H	0.34506400	1.19178000	-2.77965400
H	-0.30897300	-0.45685600	-2.81698200
C	1.91679200	2.49817500	2.18323700
C	0.67473100	1.98692500	1.74756900
C	0.27903100	1.93199400	0.37960400
F	1.94484800	4.14544500	2.50874900
H	0.85846500	2.55595600	-0.30246700
H	2.72320700	2.50397000	1.44720500
H	2.25697000	2.18651700	3.17229200
H	-0.03672100	1.64501200	2.49906300

MeSe⁻-TS-S_N2:

E = -1609.18

H = -1537.22

G = -1566.80

***N*imag** = 1, -337.42184 cm^{-1}

Se	0.34992000	-0.35245800	-0.80396800
H	1.15043200	1.06855000	3.40334900
H	-1.26724900	0.43298000	-2.65106000
C	-0.20842800	0.15665300	-2.62974100
H	0.38711700	0.99880500	-2.99663100
H	-0.05385700	-0.69654900	-3.29747100
C	-0.17376700	1.85560500	0.28431000
H	-0.59331600	0.80160600	2.83103200
F	-0.68361600	3.72625600	0.77758400
C	0.73117600	1.67090900	1.41090000
C	0.41243800	1.15041600	2.60930600
H	-1.21534300	1.59571900	0.36990900
H	0.17079400	2.32721700	-0.61730500
H	1.75360800	2.00814200	1.24395000

MeSe⁻-TS-a-S_N2':

E = -1612.61

H = -1540.63
G = -1569.58
***N*imag** =1, -165.98170 cm^{-1}

Se	0.57913100	-0.00436800	-0.67215200
H	-0.72932800	2.06464800	0.13538600
H	-1.33245100	0.81107600	-2.17978400
C	-0.33018400	0.41508800	-2.37359300
H	0.24096500	1.14594300	-2.95417000
H	-0.42267400	-0.50683700	-2.95423200
C	1.91391400	2.44331600	2.20326200
C	0.71678900	1.92168900	1.73227800
C	0.33428100	1.92894800	0.34381900
F	1.90583200	4.25948100	2.58351100
H	0.94389600	2.57144000	-0.29216700
H	2.73780300	2.57338800	1.50532000
H	2.21716300	2.21989000	3.22223500
H	-0.02083500	1.56801100	2.45276600

$MeTe^{-}$-TS-S_N2:
E = -1593.47
H = -1521.84
G = -1552.53
***N*imag** = 1, -305.30010 cm^{-1}

Te	0.32447700	-0.50794600	-0.91219200
H	1.15073700	1.11430300	3.42411500
H	-1.40046800	0.46305100	-2.82852000
C	-0.35251900	0.15979300	-2.87826800
H	0.25766400	1.00047000	-3.21595100
H	-0.24751500	-0.66825700	-3.58342100
C	-0.18295500	1.81783700	0.28997400
H	-0.59053800	0.81360200	2.86155700
F	-0.68610200	3.76852700	0.77434600
C	0.72352800	1.66193600	1.41734200
C	0.41171400	1.16532800	2.62875100
H	-1.23227200	1.59979700	0.39879300
H	0.14378400	2.32149500	-0.60114000
H	1.74285800	2.00468400	1.24245800

$MeTe^{-}$-TS-a-S_N2′:
E = -1594.74
H = -1523.17
G = -1553.46
***N*imag** =1, -82.81495 cm^{-1}

Te	0.59568700	-0.16521200	-0.76874100
H	-0.64227800	2.11364100	0.06416500
H	-1.44607900	0.74926800	-2.36301200
C	-0.45644500	0.35531700	-2.60522700
H	0.11238800	1.09669700	-3.16980000
H	-0.56621500	-0.55228200	-3.20326700
C	1.89918100	2.36955200	2.22825500

C	0.73895100	1.86998600	1.70931900
C	0.40464700	1.90464700	0.29371900
F	1.94570200	4.45484700	2.67578600
H	1.06516500	2.53635300	-0.29960500
H	2.73084600	2.63876900	1.58791400
H	2.12412700	2.27286600	3.28236800
H	-0.03274400	1.50465300	2.38748300
